# Correlated studies of photoluminescence, vibrational spectroscopy and mass spectrometry concerning the pantoprazole sodium photodegradation

**DOI:** 10.1038/s41598-022-13648-6

**Published:** 2022-06-09

**Authors:** Mihaela Baibarac, Mirela Paraschiv, Radu Cercel, Ion Smaranda, Cristina Bartha, Alexandru Trandabat

**Affiliations:** 1 National Institute of Materials Physics, Laboratory of Optical Processes in Nanostructured Materials, Atomistilor Street 405A, POB MG 7, 077125 Bucharest, Romania; 2 National Institute of Materials Physics, Laboratory of Magnetism and Superconductivity, Atomistilor Street 405A, POB MG-7, R077125, Bucharest, Romania; 3 SC Intelectro Iasi SRL, 700470 Iasi, Romania

**Keywords:** Condensed-matter physics, Materials science

## Abstract

In this work, new optical evidences concerning the changes induced of the UV light on pantoprazole sodium (PS), in solid state and as aqueous solution, are reported by UV–VIS spectroscopy, photoluminescence (PL), Raman scattering and FTIR spectroscopy. New evidences concerning the products of the PS photodegradation pathways are reported by the correlated studies of thermogravimetry and mass spectrometry. The influence of the excipients and alkaline medium on the PS photodegradation is also studied. New aspects regarding the chemical mechanism of the PS photodegradation in the presence of the water vapor and oxygen form air and the alkaline medium are shown. Our results confirm that the PS photodegradation induced of the water vapors and oxygen from air leads to the generation of 5-difluoromethoxy-3H-benzimidazole-2-thione sodium, 5-difluoromethoxy-3H-benzimidazole sodium, 2-thiol methyl-3, 4-dimethoxypyridine and 2-hydroxymethyl-3, 4-dimethoxypyridine, while in the alkaline medium, compounds of the type of the 2-oxymethyl-3,4-dimethoxypyridine sodium salts are resulted.

## Introduction

Pantoprazole sodium (PS), known under the name of protonix or 5-(difluoromethoxy)-2-(3,4-dimethoxy-2-pyridinyl)methylsulfinyl-1H-benzimidazole sodium salt (**1**) is used in the therapeutic schema for stomach ulcers and gastroesophageal reflux disease^1^. The best known of side effects of this drug are vomiting, diarrhea, headaches, joint pain and abdominal pain. All these inconvenient were induced of the degradation products of PS. In this context, a sustained effort has been focused on the determination of the impurities in drugs containing pantoprazole, including its degradation products, the most adequate methods used for this purpose being high-performance liquid chromatographic (HPLC)^2^ and UV–VIS spectroscopy^3,4^.

The instability of PS (**1**) was studied since 1999, when was demonstrated by HPLC that the degradation was dependent of the protons and salt concentration^5^. The first information concerning the PS degradation products were reported in 2013, when by the HPLC and ^13^C NMR studies was demonstrated that they correspond to 5-difluoromethoxy-3H-benzimidazole-2-thione sodium (**2**) and 2-hydroxymethyl-3, 4-dimethoxypyridine (**3**)^-^^6^. According to the spectroscopic studies reported in 2009, both UV–VIS and FTIR spectroscopy, were reported to be valuable techniques to highlight charge-transfer complexes^7^. Other detection methods used for the highlighting products of the oxidation reaction of the pantoprazole were electron paramagnetic resonance^8^ and HPLC-diode-array^9^. The reported photo-stabilization strategies of pantoprazole have involved the using polymeric microparticles^10,11^. In comparison with this progress, this work will report new evidences concerning the photodegradation process of PS (**1**) by complementary optical spectroscopy techniques such as photoluminescence (PL), UV–VIS spectroscopy, Raman scattering and FTIR spectroscopy. The role of the excipients as well as alkaline medium on the PS photodegradation process will be also analyzed. New evidences concerning the products of the PS photodegradation pathways are reported by the correlated studies of thermogravimetry and mass spectrometry.

## Results and discussion

### New evidences on photodegradation of PS (1), alone and in the presence of excipients, highlighted by PL

**Figure 1:**
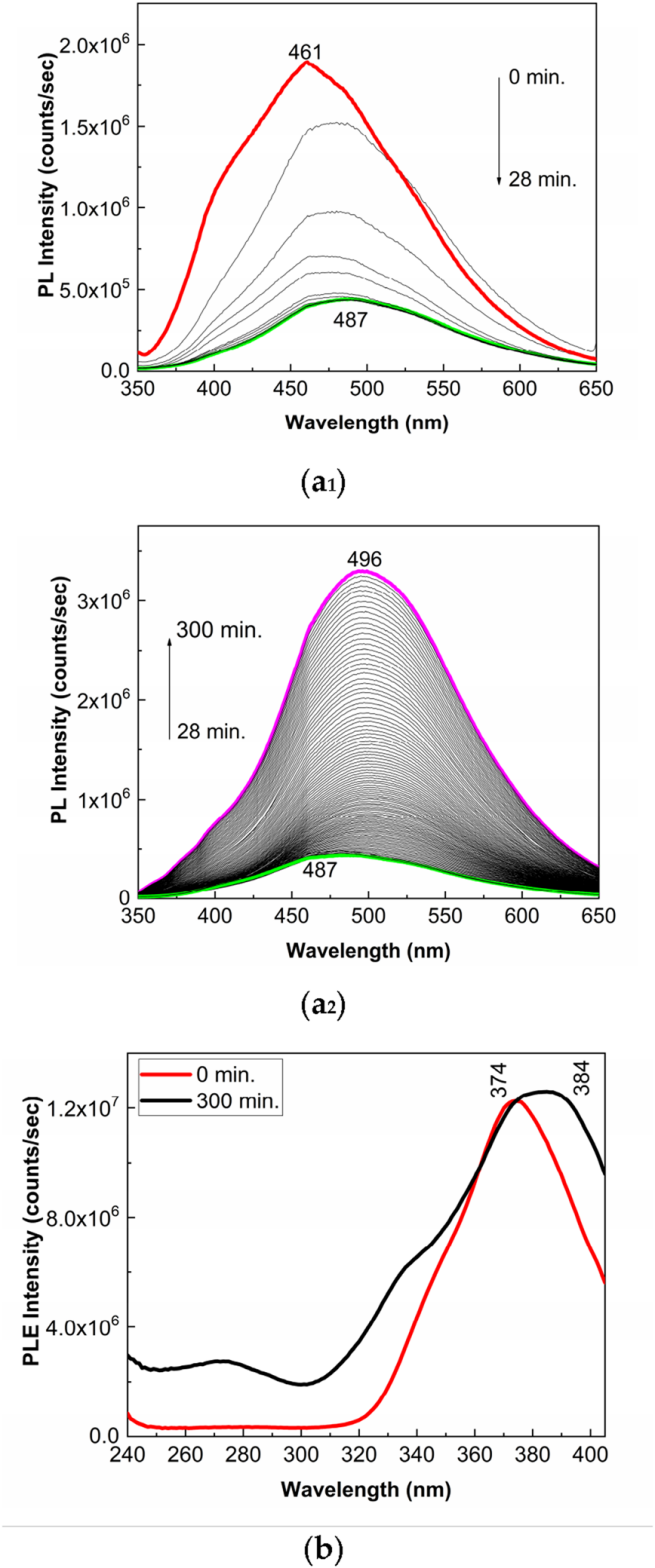
PL (**a**_**1**_ and **a**_**2**_) and PLE (**b**) spectra of PS (**1**) and their evolution when the samples are exposed 300 min. to UV light. PL and PLE spectra were recorded at the excitation emission wavelengths of 335 nm and 425 nm, respectively. Red, green, and magenta curves in (**a**_**1**_) and (**a**_**2**_) correspond to the PL spectra of PS (**1**) sample prior and after 28 min and 300 min of UV light exposure, respectively, while black curves show PL spectra successively recorded.

Figure 1a_1_,a_2_, and 1b show the PL and PLE spectra of PS (**1**) in the powder state, in dark conditions and under UV light. In the initial state, the PS (**1**) powder is characterized by: i) a PL spectrum with the maximum at 461 nm having the intensity equal to 1.89 × 10^6^ counts/sec (Fig. 1a_1_); and ii) a PLE spectrum with the maximum at 374 nm (Fig. 1b). The exposure to the UV light of the PS (**1**) induces the following changes in: i) the PL spectrum, a decrease in the intensity of the emission band from 1.89 × 10^6^ counts/sec to 3.98 × 10^5^ counts/sec in the first 28 min. (Fig. 1a_1_), the variation accompanied of a shift of the maximum of this band from 461 to 487 nm; further exposure of the PS (**1**) powder to UV light for another 272 min. induces an increase in the intensity of the PL band from 3.98 × 10^5^ counts/sec to 3.25 × 10^6^ counts/sec, simultaneous with the shift of the PL band from 487 to 496 nm (Fig. 1a_2_); and ii) the PLE spectrum, a shift of the band from 374 to 384 nm without the significant variation in the intensity (Fig. 1b). These variations indicate that the PS (**1**) photodegradation process involves two stages, the first developing in the first 28 min. and the second taking place successively in the next 272 min.

**Figure 2:**
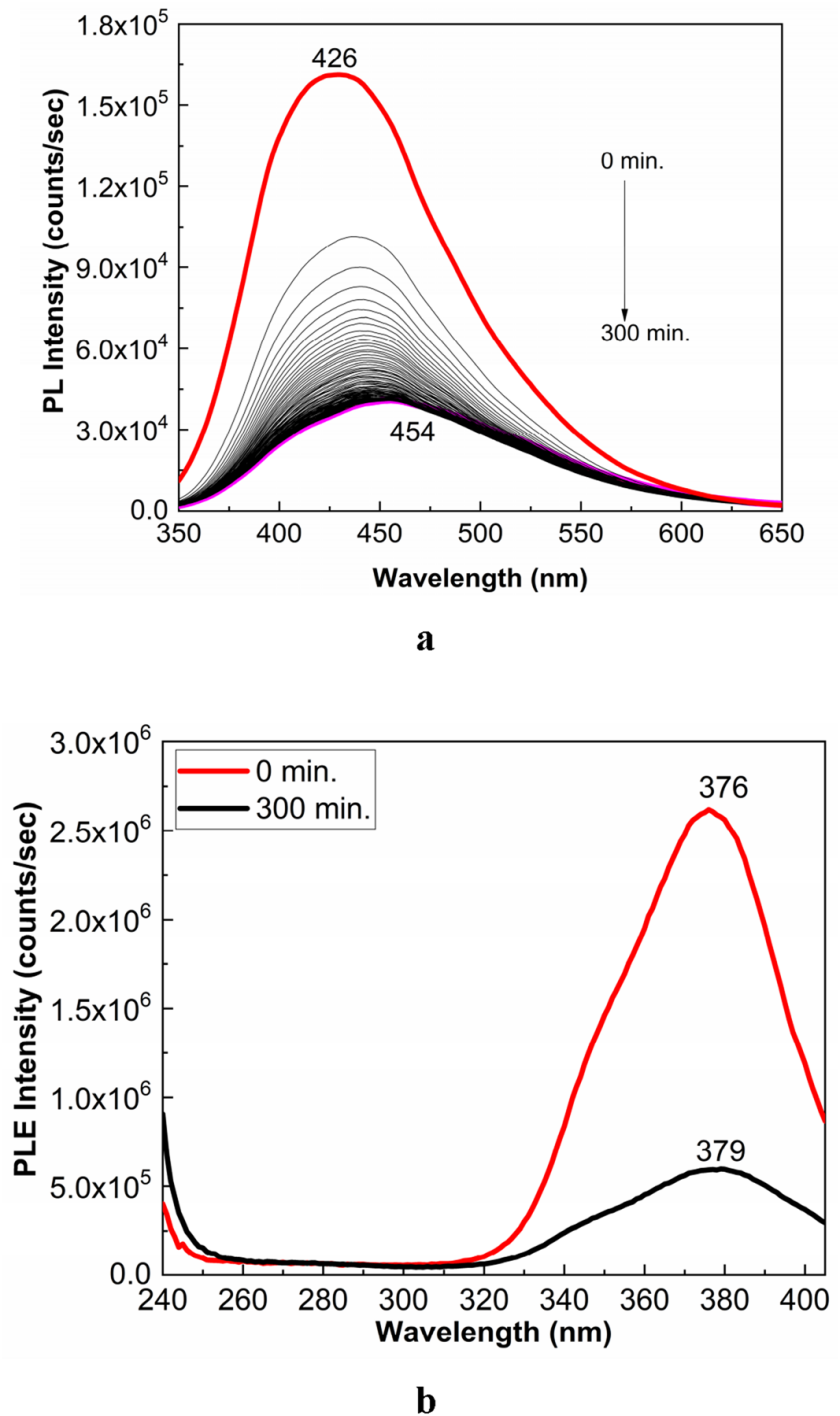
PL (**a**) and PLE (**b**) spectra of the Controloc (**4**) drug and their evolution during of 300 min. exposure to UV light. PL and PLE spectra were recorded at the excitation and emission wavelengths of 335 nm and 425 nm, respectively. In Figure (**a**), the red and magenta curves correspond to the PL spectra of the Controloc (**4**) drug prior and after 300 min of UV light exposure, while the black curves show PL spectra successively recorded.

According to Figs. 2a,b, significant changes are remarked in the PL and PLE spectra of the PS in the presence of excipients used for the drug marketed under the name of Controloc (**4**). Thus, the PL spectrum of Controloc (**4**) is characterized by an emission band with the maximum at 426 nm having the intensity equal to1.62 × 10^5^ counts/sec (Fig. 2a), while the PLE spectrum shows a band with the maximum at 374 nm (Fig. 2b). Lower intensity of the PL band in the case of Controloc (**4**) indicates that the excipients act as the PL quenching agents of PS (**1**). As the increasing of the exposure time to UV light, up to 300 min., the following changes are remarked in: i) the PL spectrum of Controloc (**4**), a gradual decrease in the intensity from 1.62 × 10^5^ counts/sec to 3.57 × 10^4^ counts, simultaneous with the shift from 426 to 454 nm (Fig. 2a); and ii) the PLE spectrum of Controloc (**4**), a significant decrease in the intensity from 2.75 × 10^6^ counts/sec to 5 × 10^5^ counts/sec, the change accompanied by the shift of the PLE band from 376 to 379 nm (Fig. 2b).

In order to explain how excipients, affect the PS PL spectrum, it is important to note that the main constituents of a tablet of Controloc are: PS, Na_2_CO_3_, mannitol, crospovidone, povidone K 90, calcium stearate, hypromellose, povidone K 25, titanium dioxide (E 171), yellow iron oxide (E172), propylene glycol, copolymer of ethyl acrylate and methacrylic acid (1: 1), polysorbate 80, sodium lauryl sulfate, triethyl citrate, shellac, iron oxide (E172), and concentrated ammonia. Polyvinylpyrrolidone (PVP) is a chemical compound commercialized under the name crospovidone, povidone K 90 and povidone K 25. The main constituents, which show photoluminescent properties are PVP, TiO_2,_ sodium lauryl sulfate (SLS), polysorbate 80 (P80) and mannitol. Suppl. Fig. S1 shows the PL spectra of PVP, TiO_2_, SLS, P80 and mannitol and their mixtures with PS. According to Suppl. Fig. S1, the PL bands of PVP, TiO_2_, SLS, P80 and mannitol, prior to the UV light exposure, are peaked at 405, 562, 435, 446 and 399 nm. The PL spectra of PS (20 mg) added by grinding at 50 mg PVP, TiO_2_, SLS, P80 and mannitol, respectively, highlight bands at 406, 485, 484, 461 and 483 nm. There results indicate that the difference between the maximum of PL band of PS (1) (Fig. 1a_1_) and Controloc (Fig. 2a), prior to exposure to UV light, origins in the presence of PVP, commercialized under the name crospovidone, povidone K 90 and povidone K 25, in the tablet of Controloc. The exposure to UV light of the PS (20 mg) added to 50 mg PVP, TiO_2_, SLS, P80 and mannitol, labeled as PS-PVP, PS-TiO_2_, PS-SLS, PS-P80 and PS-mannitol, induces a shift of PL bands from 406 nm, 485 nm, 484 nm, 461 nm and 483 nm to 442 nm, 498 nm, 488 nm, 461 nm and 491 nm, respectively, which is accompanied of changes in the intensity of PL spectra in the case: (a) PS-PVP from 3.06 × 10^6^ counts/sec to 1.17 × 10^6^ counts/sec, (b) PS-TiO_2_ from 1.41 × 10^5^ counts/sec to 9.57 × 10^5^, (c) PS-SLS from 1.12 × 10^6^ counts/sec to 4.5 × 10^5^ counts/sec, (d) PS-P80 from 5.54 × 10^5^ counts/sec to 2.71 × 10^5^ counts/sec and (e) PS-mannitol from 1.07 × 10^6^ counts/sec to 4.69 × 10^5^ counts/sec (Suppl. Fig. S1). The lower values of PL spectrum intensity of the PS-TiO_2_, PS-SLS, PS-P80 and PS-mannitol samples in comparison with those reported in the case of PS (Fig. 1a_1_) indicates clearly that the following compounds TiO_2_, SLS, P80 and mannitol have the role of PS PL quenching agents. The adding PS (20 mg) by grinding in a matrix which no show luminescent properties, such as Na_2_CO_3_ (50 mg) induced, under UV light exposure for 300 min, a decrease in PL band intensity from 1.2 × 10^6^ counts/sec to 2.97 × 10^5^ counts/sec, simultaneously with its shift from 462 to 487 nm. This fact has evidenced that Na_2_CO_3_ has the role of PS PL quenching agent.

**Figure 3:**
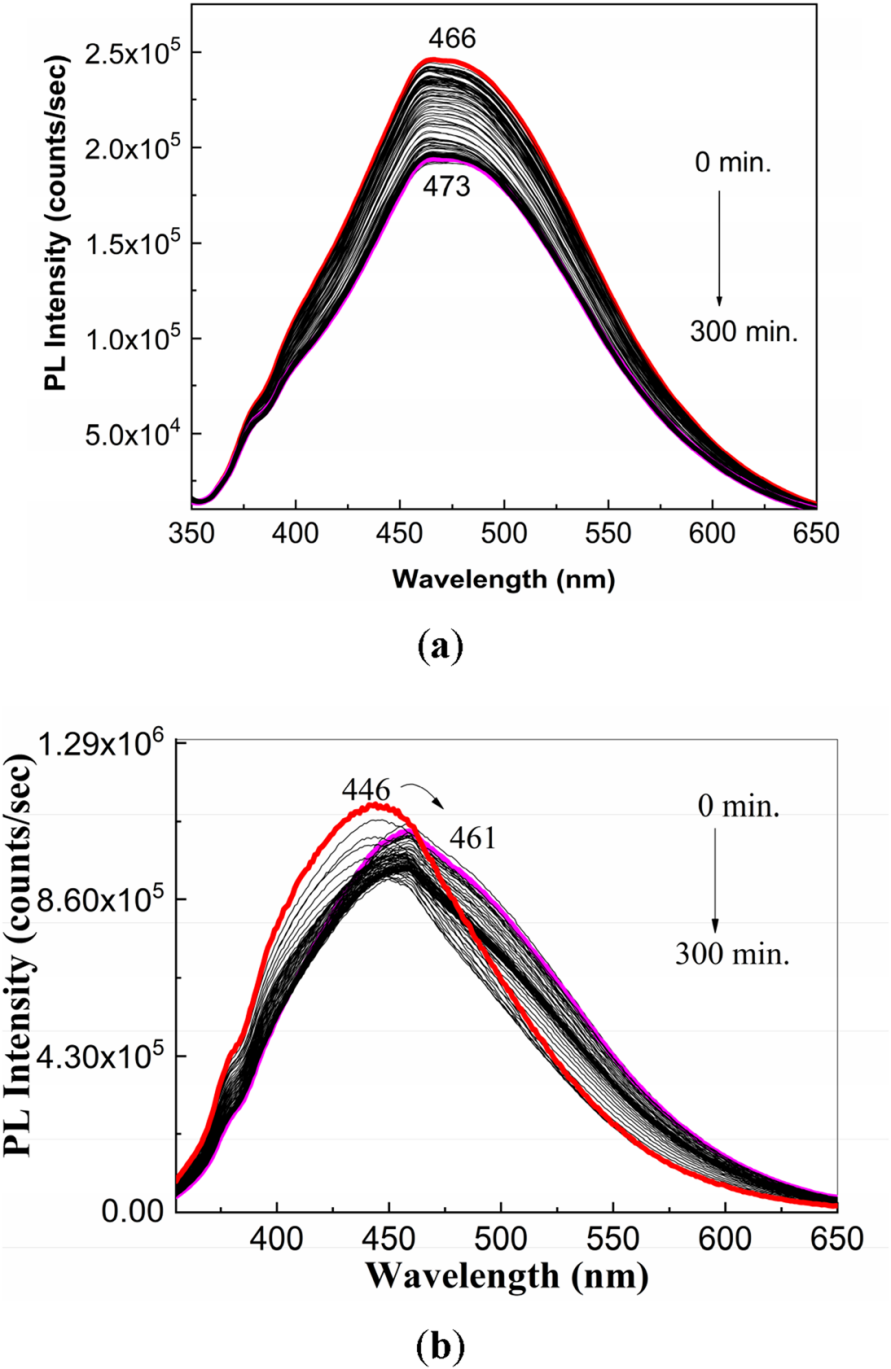
PL spectra of the aqueous solution of PS (**1**, **a**) and the Controloc drug (**4**, **b**), when the two samples are exposed to the UV light, for 300 min. The PS concentration in the two aqueous solutions was of 2 mg/ml. All PL spectra were recorded at the excitation wavelength of 335 nm. Red and magenta curves correspond to PL spectra of PS and Controloc, prior and after 300 min of UV light exposure, while the black curves show PL spectra successively recorded for the two compounds.

The PL spectrum of the PS (**1**) aqueous solution (Fig. 3) shows an emission band with the maximum at 466 nm, while the PL spectrum of the Controloc (**4**) aqueous solution shows an emission band with the maximum at 446 nm. A change in the position of the maximum of emission band of the PS (**1**) aqueous solution during the exposure to UV light from 466 to 473 nm (Fig. 3a) is reported; a similar behavior occurs in the case of the Controloc (**4**) aqueous solution, when one observes a shift of the emission band from 446 to 461 nm (Fig. 3b), the value which is no far of the emission band of the drug in powder state after the exposure to UV light (Fig. 2a). These facts allow us to invoke that the photodegradation process of the PS (**1**) powder, highlighted in Fig. 1, origins in photochemical reaction of PS (**1**) with the water vapors and the oxygen from air.

**Figure 4:**
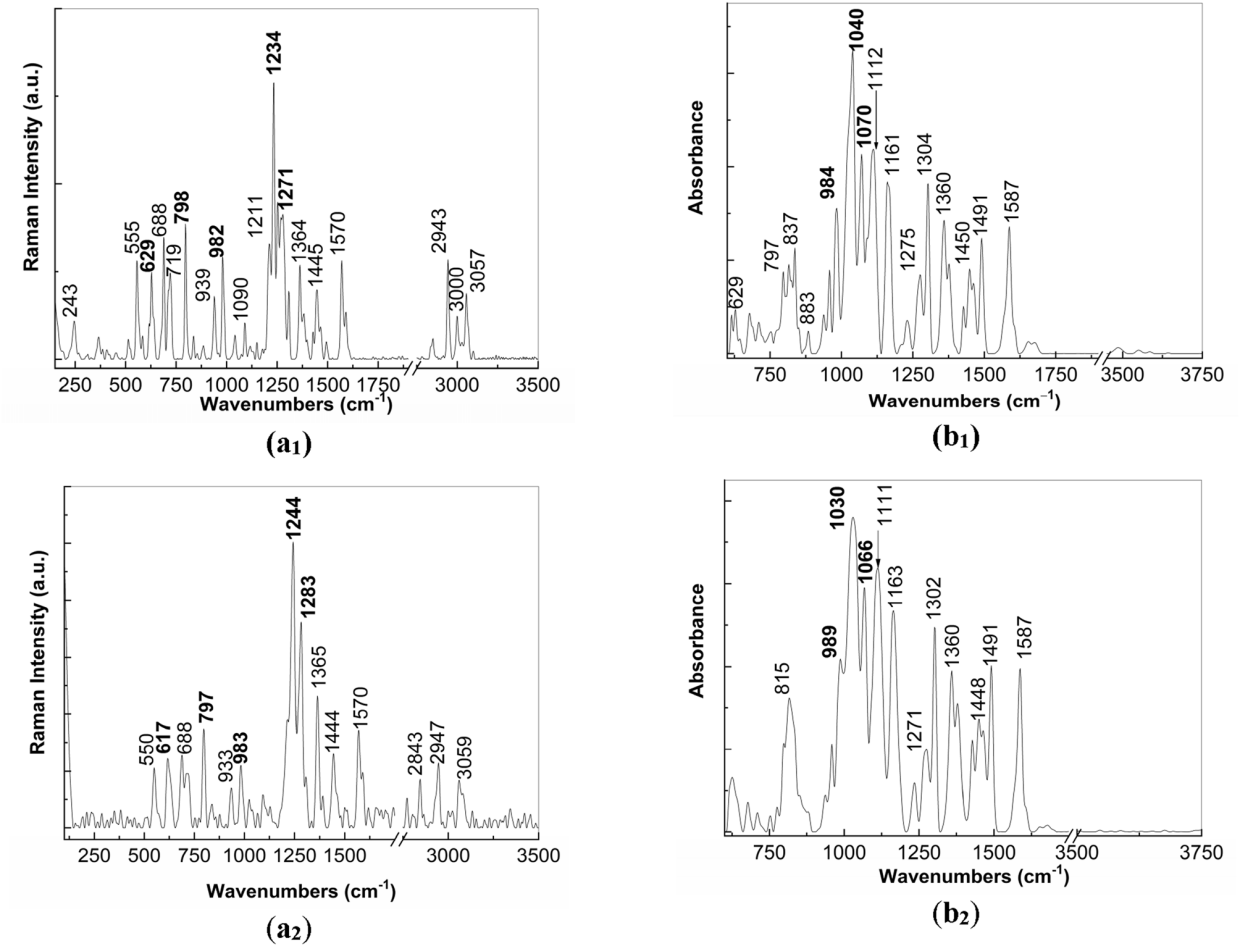
Raman (**a**) and IR (**b**) spectra of PS (**1**) in powder state prior (**a**_**1**_**, b**_**1**_) and after 300 min. exposure to UV light (**a**_**2**_**, b**_**2**_).

**Scheme 1:**
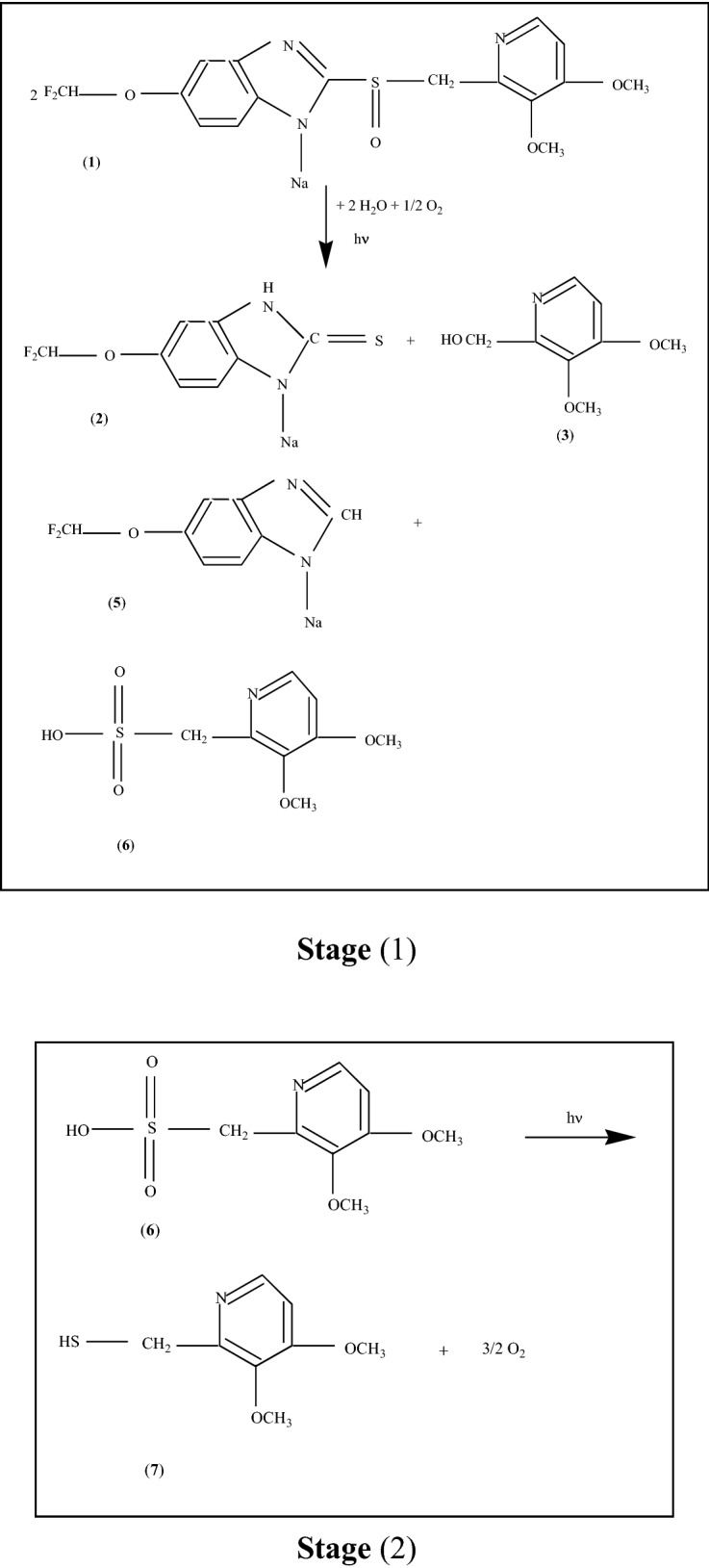
The photodegradation reactions of PS (**1**) in the presence of the H_2_O vapors and O_2_ from air.

In order to understand the changes induced to PS (**1**), Fig. 4 shows the Raman and IR spectra of PS (**1**) in powder state, before and after the exposure to the UV light. The Raman spectrum of PS (**1**, Fig. 4a1) shows the lines with peaks at 243—555, 629, 688, 719, 798, 939, 982, 1090, 1211, 1234, 1271, 1308, 1364, 1445, 1570, 2943, 3000 and 3057 cm^−1^, whose assignment is presented in Suppl. Table S1^12,13^. According to Fig. 4b_1_, the main IR bands of PS are shown in Suppl. Table S2^12,13^. After the exposure to UV light of PS in powder state time of 300 min., Fig. 4a_2_,b_2_ highlight the following changes in: i) the Raman spectra, the ratio between the intensities of Raman line peaked at 980 and 797 cm^−1^ (I_980_/I_797_) decreases from 0.75 to 0.63 and a shift of the Raman lines from 629, 1234, 1271 cm^−1^ to 617, 1244 and 1283 cm^−1^, respectively, occurs; and ii) IR spectra, a shift of the IR bands from 984, 1040 and 1070 cm^-1^ to 989, 1030 and 1066 cm^−1^, respectively, simultaneously with the decrease in the absorbance of the IR band peaked at 1040–1030 cm^−1^ take place. The variation of the I_980_/I_797_ ratio and the decrease in the absorbance of the IR band peaked at 1040–1030 cm^−1^, attributed to the vibrational mode of stretching OC + stretching SO + in plane bending OSC, indicate that the PS photodegradation under UV light involves a broken of the CSCH bond and the transformation of OSC bond in other bonds as those reported in Ref.^6^. To understand these changes, Scheme 1 shows the photodegradation reactions of PS.

According to Scheme 1, (i) the photodegradation products of Stage 1 are 5-difluoromethoxy-3H-benzimidazole-2-thione sodium (**2**) and 2-hydroxymethyl-3, 4-dimethoxypyridine (**3**), 5-difluoromethoxy-3H-benzimidazole sodium (**5**) and 3, 4-dimethoxy-2-pyridyl methanesulfonic acid (**6**); and (ii) the reaction product of the Stage 2 is 2-thiomethyl-3, 4-dimethoxypyridine (**7**).

The PL spectrum of the PS powder is one complex which can be deconvoluted in three emission bands peaked at 2.36 eV (~ 525 nm), 2.68 eV (~ 462 nm) and 3.05 eV (~ 406 nm) (Suppl. Fig. S2), which were assigned to the luminescence centers from the n-π* and π–π* transitions of the benzimidazole units (C_6_H_3_N_2_(Na)C-)^14,15^ and methoxy-pyridine entity (C_5_NH_2_(OCH_3_)_2_CH_2_-)^16^, respectively. The PL bands of PS, prior to the UV light exposure peaked, at 2.36, 2.68 and 3.05 eV have the intensity equal to 7.8 × 10^5^, 1.48 × 10^6^ and 9.54 × 10^5^ counts/sec (Suppl. Fig. S2a). After 300 min of UV light exposure, the intensities of the three PL bands at 2.36, 2.68 and 3.05 eV are equal to 3.09 × 10^5^, 2.65 × 10^5^ and 7.22 × 10^4^ counts/sec (Supp. Fig. S2b). These values indicate a diminution in the intensity of the PL bands at 2.36, 2.68 and 3.05 eV of ~ 2.52, 5.58 and 13.21 times. These variations can be explained if we take into account Scheme 1S, when PS is transformed in new compounds containing benzimidazole units, as in 5-difluoromethoxy-3H-benzimidazole-2-thione sodium (**2**) and 5-difluoromethoxy-3H-benzimidazole sodium (**5**), and methoxy-pyridine entity like in 2-hydroxymethyl-3, 4-dimethoxypyridine (**3**) and 2-thiomethyl-3, 4-dimethoxypyridine (**7**). In this stage of our studies, we are tempted to assign the red shift to the new groups existing in photodegradation products, i.e. OH in -hydroxymethyl-3, 4-dimethoxypyridine (**3**), CH in 5-difluoromethoxy-3H-benzimidazole sodium (**5**), SH in 2-thiomethyl-3, 4-dimethoxypyridine (**7**) and C=S in 5-difluoromethoxy-3H-benzimidazole-2-thione sodium (**2**). The groups OH, SH and CH are known as electron donating groups, which have as effect of increasing electron density in the molecule, while the C=S group is known as an electron withdrawing group, its effect being reported to reduce electron density in a molecule. As recently reported, the presence of the electron acceptor and/or donor groups into conjugated chromophores induce the red-shifted emissions^17^.

Returning to Fig. 3, at the end of the 300 min of irradiation of the aqueous solutions of PS and Controloc, the two solutions are light brown and the formation of a brown precipitate also takes place. Among the photodegradation products mentioned above, water-soluble compounds are 5-difluoromethoxy-3H-benzimidazole-2-thione sodium (**2**) and 5-difluoromethoxy-3H-benzimidazole sodium (**5**), while compounds 2-hydroxymethyl-3, 4-dimethoxypyridine (**3**) and 2-thiomethyl-3, 4-dimethoxypyridine (**7**) are insoluble in water. Suppl. Fig. S3 shows IR spectra of the compounds insoluble and soluble in water. According to Suppl. Fig. S3a, the IR spectrum of the water-soluble compounds shows bands peaked at 987, 1074, 1105, 1163 and 1589 cm^−1^, which are very close of IR bands at 984, 1070, 1112, 1161 and 1587 cm^−1^ (Fig. 4b_1_), assigned to the vibrational modes of stretching CH + torsion CSCN, stretching CF, out-of-plane OCFF, in plane bending NCH + HCC and in plane bending CCN, respectively^12,13^. The IR bands at 1639 and 3437 cm^−1^ are assigned to the OH bending^18^ and OH stretching^19^ vibrational modes. According to Scheme 1, the presence of these vibrational modes there is only in compounds 5-difluoromethoxy-3H-benzimidazole-2-thione sodium (**2**) and 5-difluoromethoxy-3H-benzimidazole sodium (**5**). In the case of the IR spectrum of the water-soluble compounds are observed the band peaked at 987, 1080, 1639 and 3458 cm^−1^ (Suppl. Fig. S3b) assigned to the vibrational modes of stretching CH + torsion CSCN, C–O stretching in phenol ring^19^, OH bending^18^ and OH stretching^19^ vibrational modes. These IR bands indicates that water-insoluble compounds are 2-hydroxymethyl-3, 4-dimethoxypyridine (**3**) and 2-thiomethyl-3, 4-dimethoxypyridine (**7**). In order to highlighted the contribution of the compounds soluble and insoluble in water on red-shift of the emission bands, the PLE and PL spectra of the photodegradation products soluble and insoluble, respectively, in water, are shown in Suppl. Fig. S4. Thus, in Suppl. Fig. S4 one observes that (i) the mixture based on 2-thiomethyl-3, 4-dimethoxypyridine (**7**) and 2-hydroxymethyl-3, 4-dimethoxypyridine (**3**) show in PLE spectrum a band at 316 nm which is accompanied of a shoulder at 370 nm and in PL spectrum a band at 498 nm; and (ii) the mixture consisting of 5-difluoromethoxy-3H-benzimidazole-2-thione sodium (**2**) and 5-difluoromethoxy-3H-benzimidazole sodium (**5**) shows in PLE spectrum a band at 369 nm and in PL spectrum a band at 472 nm. Taking into account the maximum of PL band of the compounds soluble and insoluble in water, the red shift of emission band is induced of the following two compounds 2-thiomethyl-3, 4-dimethoxypyridine (**7**) and 2-hydroxymethyl-3, 4-dimethoxypyridine (**3**).

**Figure 5:**
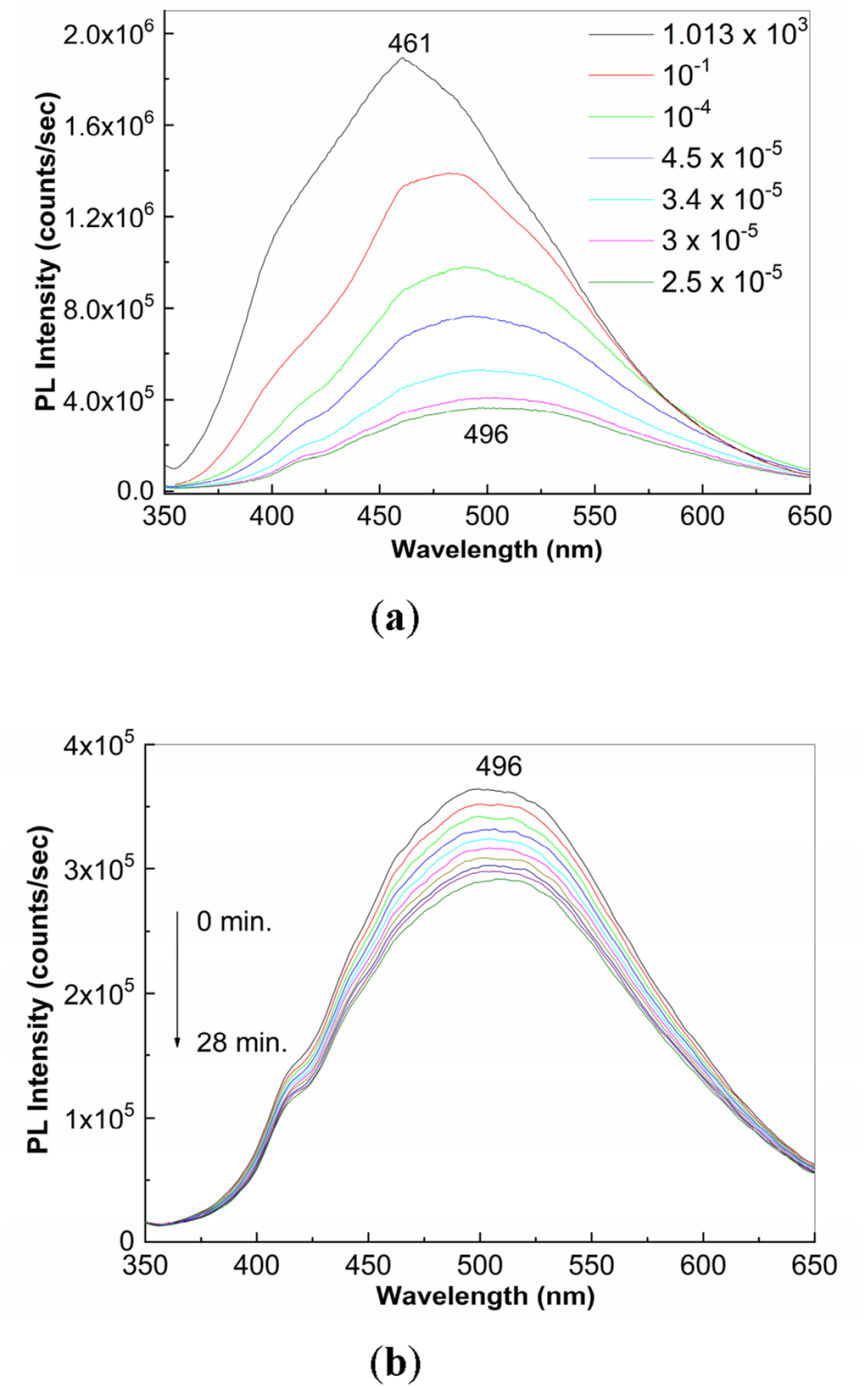
The dependence of the PL spectra of PS (**1**) depending on the pressure of environmental conditions (**a**) and the evolution of the PL spectra of PS (**1**) under vacuum conditions, when the pressure was equal to 2.5 × 10^–5^ mbar and the sample was exposed to UV light, time of 28 min. (**b**). In Figure **a**, the black, red, green, blue, cyan, magenta and olive curves correspond to the PL spectra of PS when the sample is at a pressure equal to 1.013 × 10^3^ mbar, 10^–1^ mbar, 10^–4^ mbar, 4.5 × 10^–5^ mbar, 3.4 × 10^–5^ mbar, 3 × 10^–5^ mbar and 2.5 × 10^–5^ mbar, respectively. In Figure **b**, the black, red, green, blue, cyan, magenta, dark yellow, navy, purple and olive curves correspond to the exposure times of PS to UV light equal to 164 s, 328 s., 492 s, 656 s, 820 s, 984 s, 1148 s, 1312 s, 1476 s and 1680 s.

In order to highlight the behavior of PS (**1**) exposed to the UV light in the presence of a significant diminution of the water vapors and oxygen from air, Fig. 5 show the dependence of the PL spectra of PS (**1**) depending on the pressure of environmental conditions (1.013 × 10^3^ mbar to 2.5 × 10^–5^ mbar) and the evolution of the PL spectra of PS under vacuum conditions, when the pressure was equal to 2.5 × 10^–5^ mbar and the sample was exposed to UV light. Figure 5a reveals that under the vacuum condition, by the change of the pressure form 1 atm (1.013 × 10^3^ mbar) up to 2.5 × 10^–5^ mbar, induces a significant diminution of the intensity of the PL spectra of PS (**1**) from 1.89 × 10^6^ counts/sec to 3.63 × 10^5^ counts/sec. A careful analysis of Fig. 5b evidences that at the pressure of 2.5 × 10^–5^ mbar, the exposure to UV light of PS (**1**), time of 28 min. induces a decrease in the intensity of PL from 3.63 × 10^5^ counts/sec to 2.84 × 10^5^ counts/sec, namely of ~ 1.27 times. This value it is significantly lower than that reported in Fig. 1a, when a decrease of 4.74 times of the PL intensity was reported after the exposure of PS (**1**) to UV light, time of 28 min., in the presence of water vapors and oxygen from air.

In order to highlight the effect of water vapors and oxygen, respectively, on the photodegradation process, the PL spectra of the PS powder in synthetic air (max. 20% O_2_ and min.80% N_2_) or in Ar containing ≤ 0.5 ppm O_2_ and ≤ 0.5 ppm H_2_O are shown in Suppl. Fig. S5. Regardless that PS powder is storage in synthetic air or Ar, in Suppl. Fig. S5 one observes:

(i) prior to the UV light exposure, the PL spectra of PS in Suppl. Fig. S5a_3_ and S5b_3_ highlights three emission bands peaked at 3.11, 2.65 and 2.36 eV; the three band of the PL spectrum of PS recorded in air condition (Suppl. Fig. S2a) have intensity equal to 9.54 × 10^5^ counts/sec, 1.48 × 10^6^ counts/sec and 7.8 × 10^5^ counts/sec. Significant variations in the intensities of the three emission bands at 3.11, 2.65 and 2.36 eV occur when PS PL spectrum is recorded in the presence of synthetic air and Ar. Prior to exposure to UV light, the intensity of the emission bands at 3.11, 2.65 and 2.36 eV is equal to: a) 4.19 × 10^5^ counts/sec, 2.86 × 10^5^ counts/sec and 2.16 × 10^5^ counts/sec in the case of PS storage in synthetic air (Suppl. Fig. S5a_3_); and b) 5.09 × 10^5^ counts/sec, 3.14 × 10^5^ counts/sec and 2.14 × 10^5^ counts/sec in the case of PS storage in Ar (Suppl. Fig. S5b_3_);

(ii) after exposure to UV light of the PS samples, an additional decrease in the intensities of the three bands at 3.11, 2.65 and 2.36 eV in the first 48 min and 23 min, respectively, takes place to: a) 2.75 × 10^5^ counts/sec, 6.74 × 10^4^ counts/sec and 9.2 × 10^4^ counts/sec in the case of PS storage in synthetic air (Suppl. Fig. S5a_4_); and b) 3.49 × 10^5^ counts/sec, 7.78 × 10^4^ counts/sec and 7.1 × 10^4^ counts/sec in the case of PS storage in Ar (Suppl. Fig. S5b_4_); the UV light exposure of the PS samples storage in synthetic air and Ar, more 48 min and 23 min, induces an increase in the intensity of the PL bands at 3.11, 2.65 and 2.36 eV more important for the last two, as follows: a) 3.28 × 10^5^ counts/sec, 2.32 × 10^5^ counts/sec and 2.77 × 10^5^ counts/sec in the case of PS storage in synthetic air (Suppl. Fig. S5a_5_); and b) 3.89 × 10^5^ counts/sec, 2 × 10^5^ counts/sec and 2.43 × 10^5^ counts/sec in the case of PS storage in Ar (Suppl. Fig. S5b_5_). The more pronounced increase in the intensity of the IR bands at 2.36 and 2.68 eV is caused by self-aggregation process which is induced of π − π stacking interactions and hydrogen bonds specific to benzimidazole-containing compounds^20^. Summarizing variations shown in Suppl. Fig. S5, these once again prove the interaction of PS with oxygen and traces of water.

### The influence of the buffer phosphate solution pH on the photodegradation of PS, highlighted by UV–VIS spectroscopy and PL

**Figure 6:**
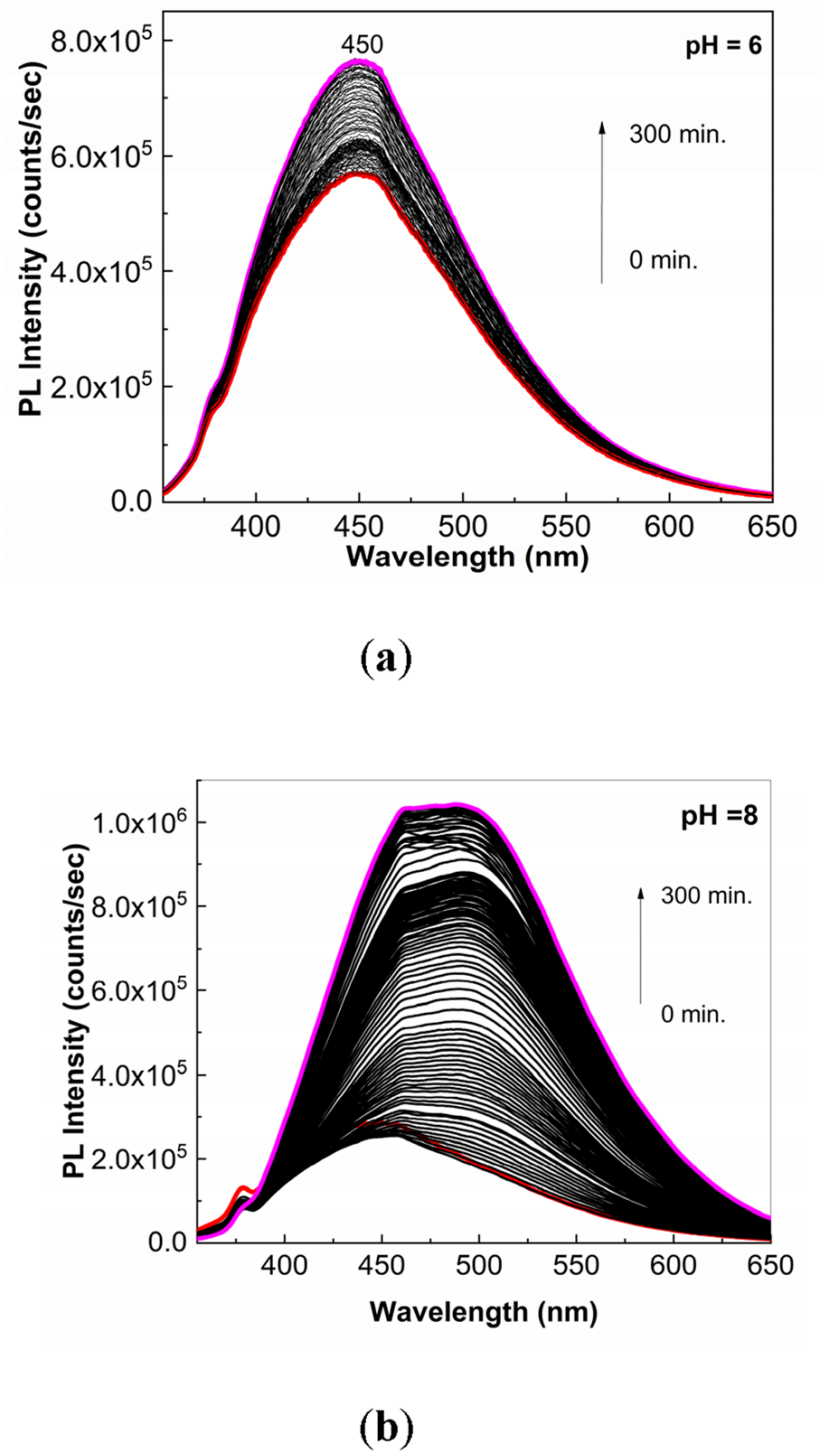
The PL spectra of PS (**1**; 2 mg/ml) in BP with the pH = 6 (**a**) and 8 (**b**) and their evolution during the 300 min. exposure to UV light. Red and magenta curves corresponds to PL spectra prior and after 300 min of UV light exposure, while the black curves show PL spectra successively recorded.

**Figure 7:**
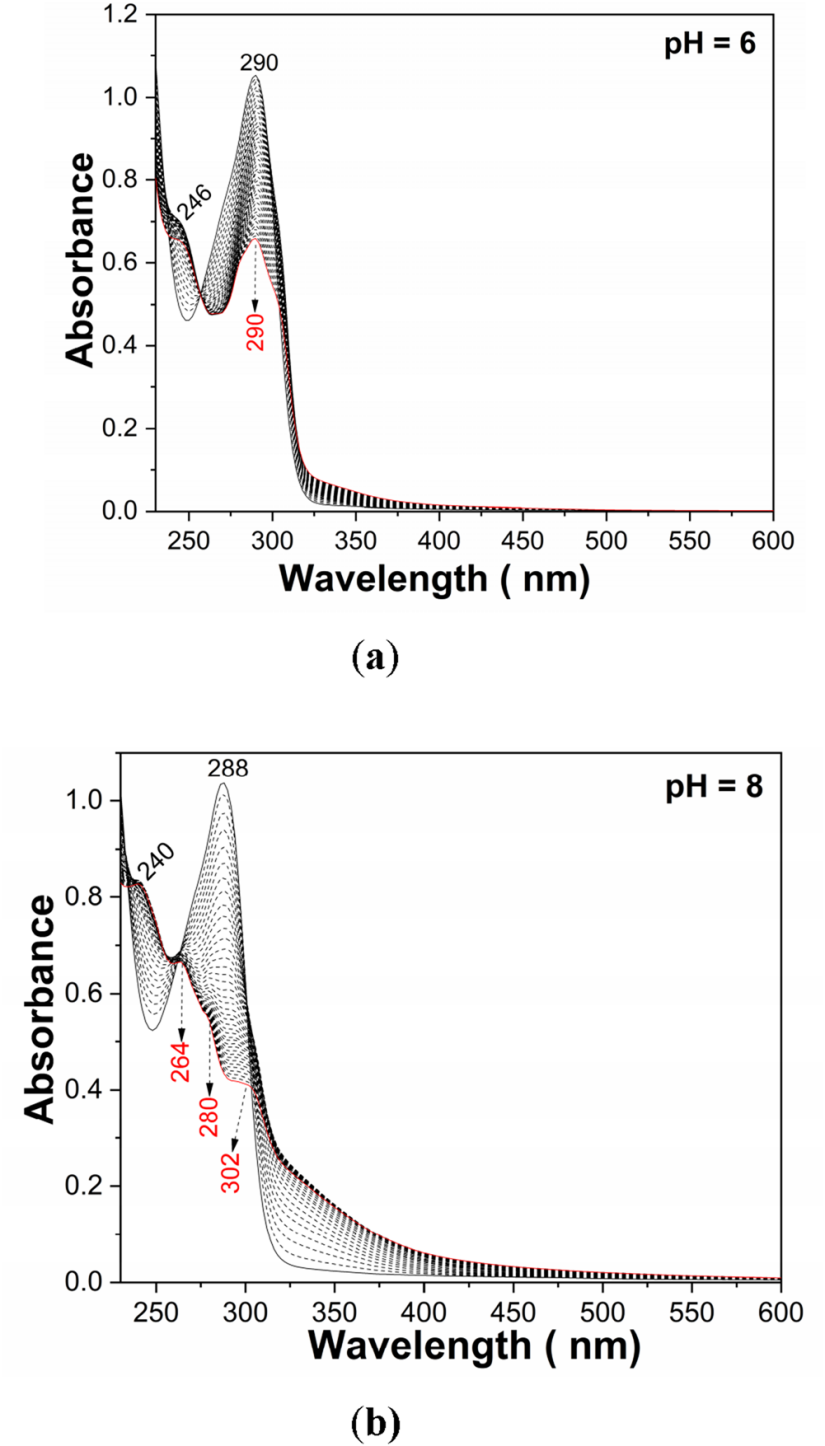
The UV–VIS spectra of the solution of PS (**1**; 2 mg/ml) in BP with the pH = 6 (**a**) and 8 (**b**) and their evolution during the 80 min. exposure to UV light. Black and red solid lines correspond to the UV–VIS spectra of the PS (**1**) solutions prior and after exposure to UV light, respectively, time of 80 min. Black dash lines correspond to the UV–VIS spectra of the PS (**1**) solution subsequently exposed for 2 min. to UV light.

**Figure 8:**
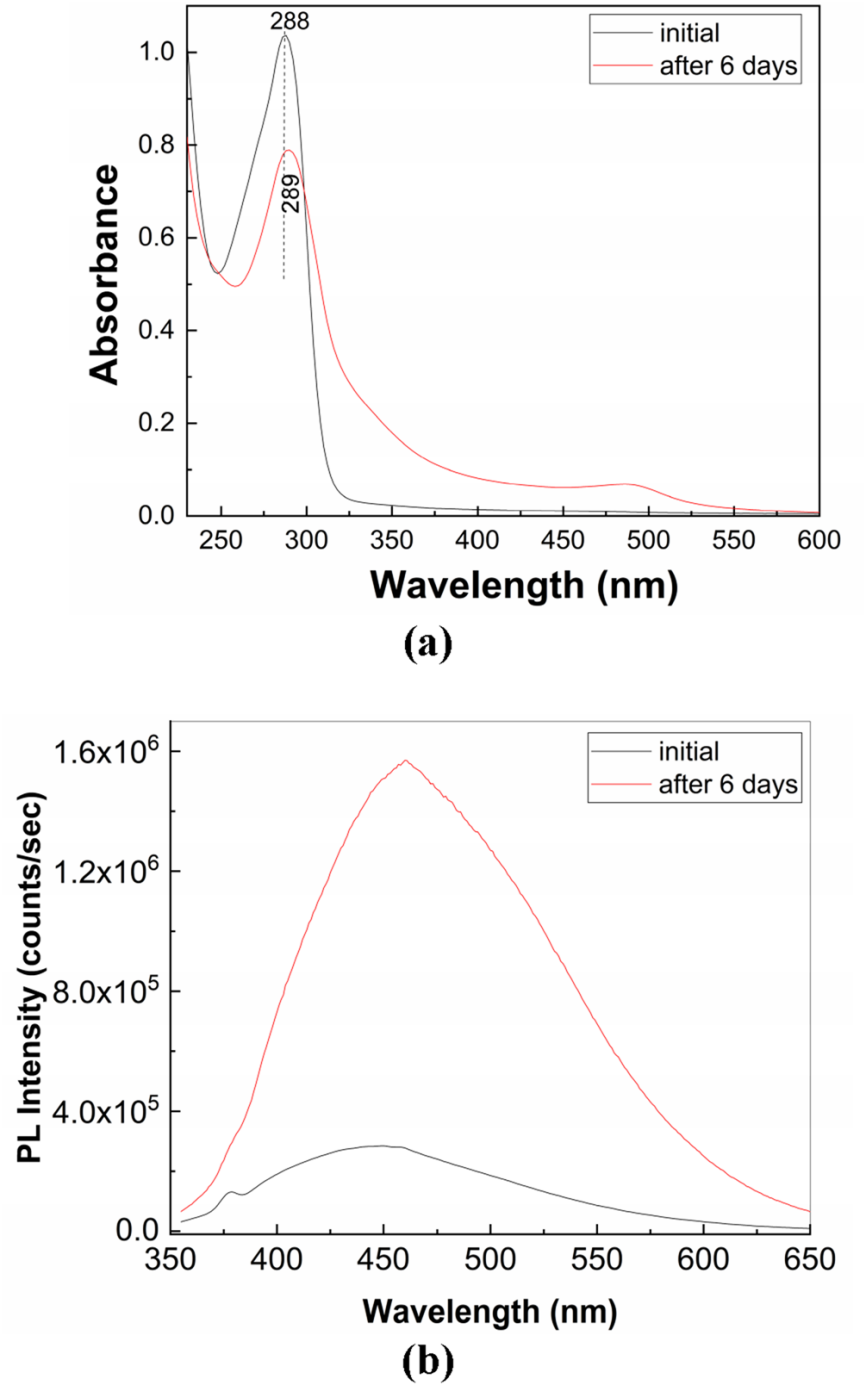
The UV–VIS (**a**) and PL (**b**) spectra of the solution of PS (1; 2 mg/ml) in BP with the pH = 8 and their evolution after the storage time of 6 days. Black and red solid lines correspond to the UV–VIS and PL spectra of the PS (1) solutions prior and after the storage time of 6 days.

Figure 6 shows PL spectra of the solution of PS (**1**) in buffer phosphate (BP) with pH equal to 6 and 8. Regardless of the PB pH value, before to exposure to UV light of the PS (**1**) solution, the PL spectra show a band with the maximum at 450 nm. In the dark conditions, the PL band intensity of PS (**1**) in BP with pH = 6 and 8 is equal to 5.71 × 10^5^ counts/sec and 2.85 × 10^5^ counts/sec, respectively. The exposure of PS (1) in BP solution with pH = 6 and 8 to UV light for 300 min. induce an intensity growth of PL band up to 7.64 × 10^5^ counts/sec and 1.04 × 10^6^ counts/sec, respectively. Significant modifications are also observed in Fig. 7, which shows the UV–VIS spectra of solution of PS (**1**; 2 mg/ml) in BP with the pH equal to 6 and 8 as well as their evolution during the exposure to UV light. The UV–VIS spectra of PS (**1**) in BP with pH = 6 and 8 are characterized of a band with the maximum at 290 and 288 nm, respectively, belonging to the C=N chromophore group^21^. The subsequently exposure of PS (**1**) in BP to UV light induces for sample with: (i) pH = 6, a progressively absorbance decrease of the band at 290 nm and the emergence of another band at 246 nm and an isosbestic point at 256 nm; and (ii) pH = 8, an absorbance diminution of the band at 288 nm of ~ 52% which occurs with its shift at 280 nm, the emergence of new bands localized in the 200–250 and 325–375 nm spectral ranges and two isosbestic points at 264 and 302 nm. These experimental facts indicate that regardless of the BP pH value, a photodegradation of PS (**1**) takes place. As shown below, the increase the BP pH value up to ~ 13.2 determines an additional absorbance diminution of the band at 288–290 nm. To exemplify the relevance of these studies in storage conditions of natural light, Fig. 8 shows UV–VIS and PL spectra prior and after storage for 6 days of the solution of PS in PB (pH = 8). The changes observed in Fig. 8 are close of those presented in Figs. 6b and 7b, they highlighting an absorbance decrease of band at 288 nm (Fig. 8a) and an intensity growth of the PL up to 1.57 × 10^6^ counts/ sec (Fig. 8b), respectively. These facts indicate clearly that the PS photodegradation takes place also in daily storage conditions in the presence of natural light.

### The NaOH influence on the PS and Controloc drug photodegradation, highlighted by PL

**Figure 9:**
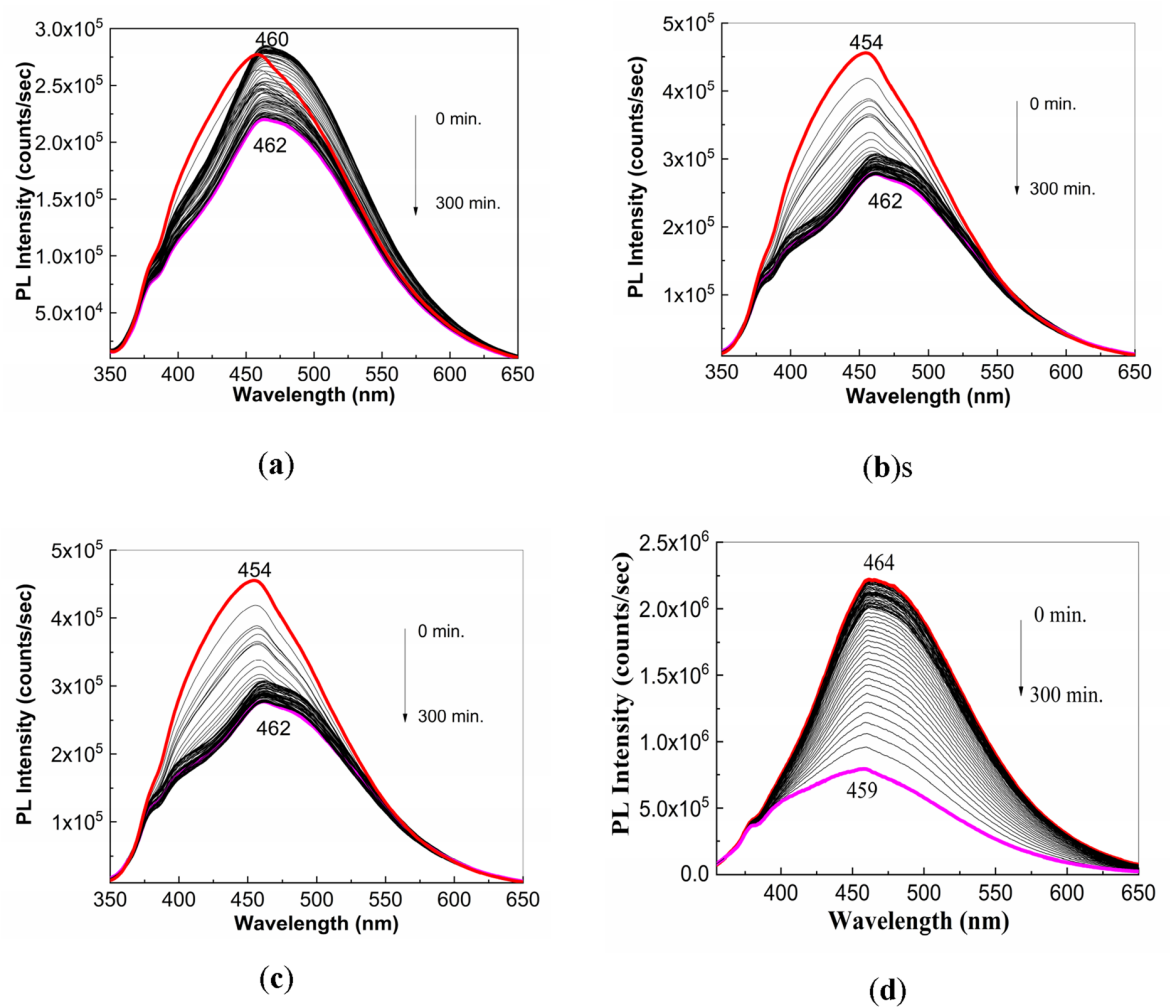
PL spectra of PS (**1**; 2 mg/ml) interacted with NaOH (0.3 M) when the volumetric ratio is equal to 2:1 (**a**), 1.5:1.5 (**b**) and 1:2 (**c**). Figure **d** shows the PL spectra of the Controloc (**4**) drug (having PS concentration of 2 mg/ml) interacted with the NaOH 0.3 M, when the volumetric ratio of the two solutions is of 2:1 and the sample is exposed to UV light, for 300 min. The red and magenta curves correspond to PL spectra of samples, prior and after to 300 min od UV light exposure, while the black curves show PL spectra successively recorded.

**Figure 10:**
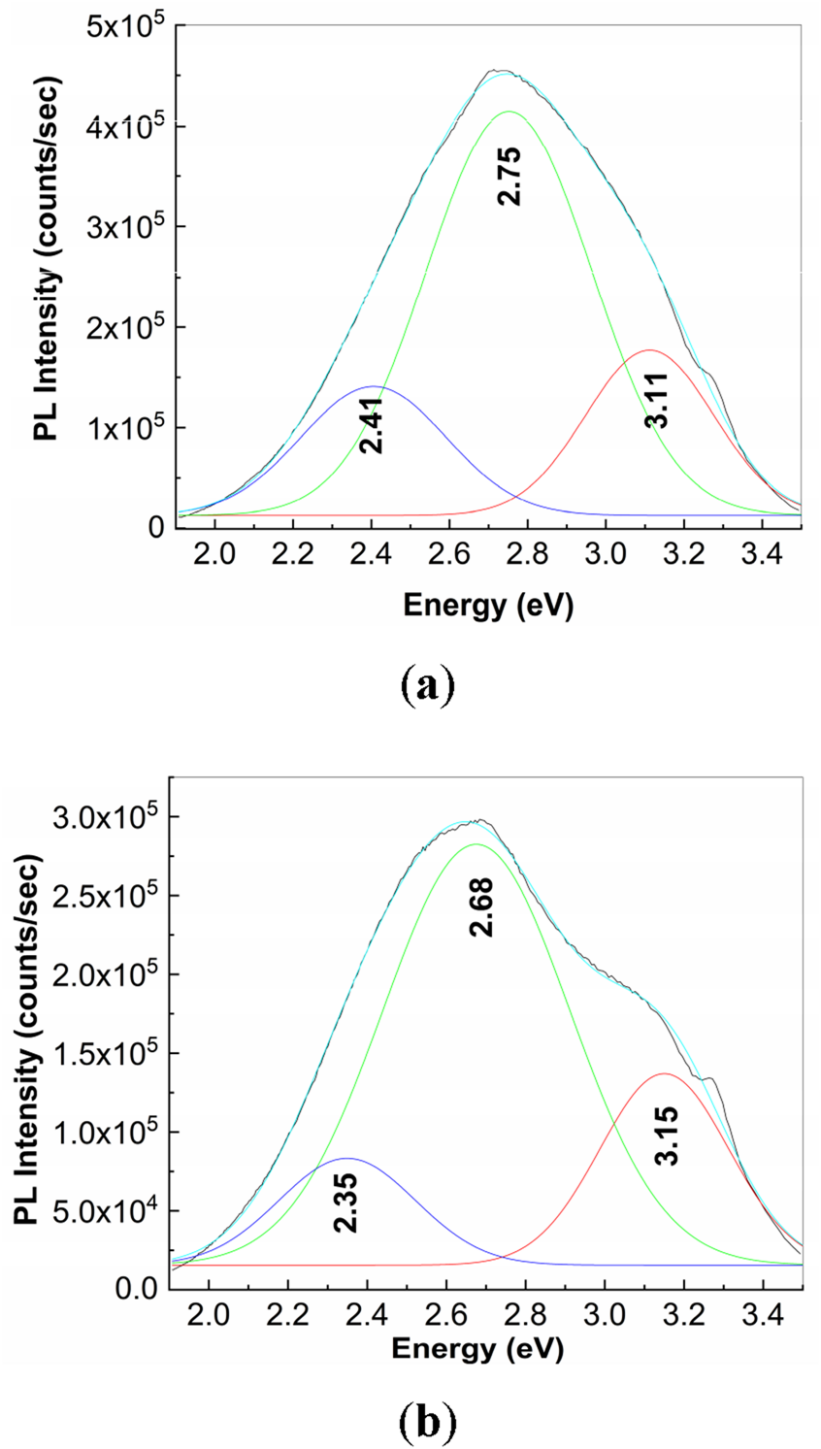
PL spectra of the sample resulted by the reaction of 1 ml PS (**1**) 2 mg/ml with 2 ml NaOH 0.3 M prior (**a**) and after the 300 min. exposure to UV light (**b**).

**Figure 11:**
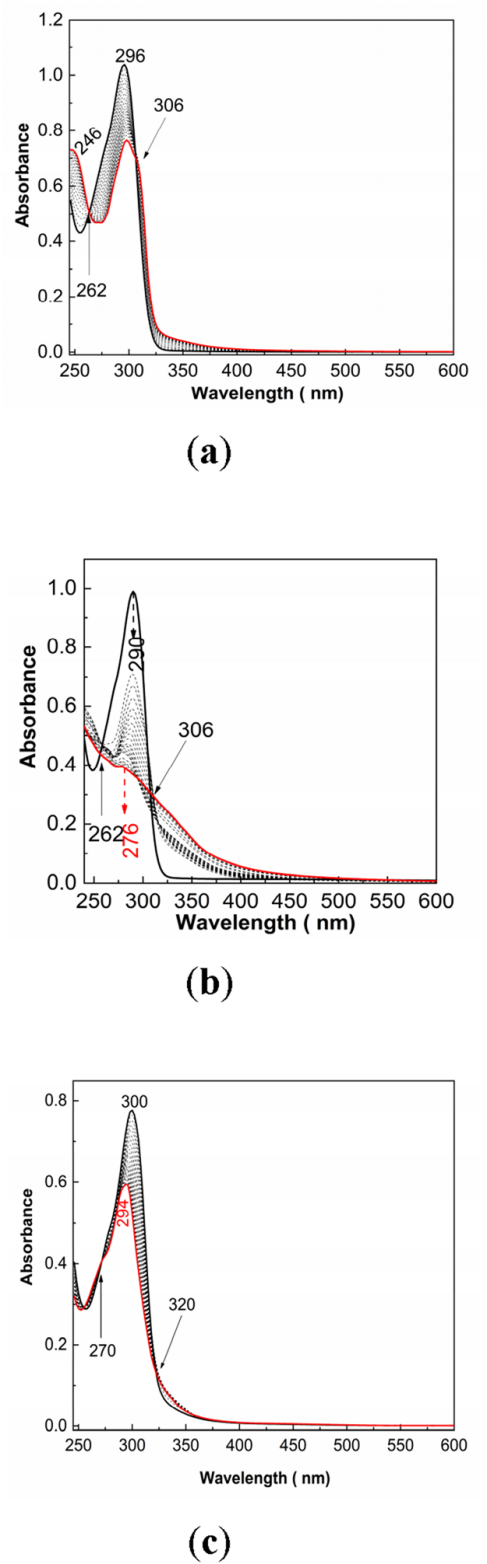
UV–VIS spectra of the PS (**1**; **a,** 0.02 mg/ml) and Controloc (**4**; **b,** the PS concentration is 0.02 mg/ml) and their evolution when the two samples are exposed 80 min. to UV light. Figure (**c**) shows UV–VIS spectra of the PS (**1**) interacted with NaOH 0.3 M. Black and red solid lines correspond to the UV–VIS spectra of the PS (**1**) and Controloc (**4**) solutions prior and after exposure to UV light, respectively, time of 80 min. Black dash lines correspond to the UV–VIS spectra of the PS (**1**) and Controloc (**4**) solutions subsequently exposed for 2 min. to UV light.

**Figure 12:**
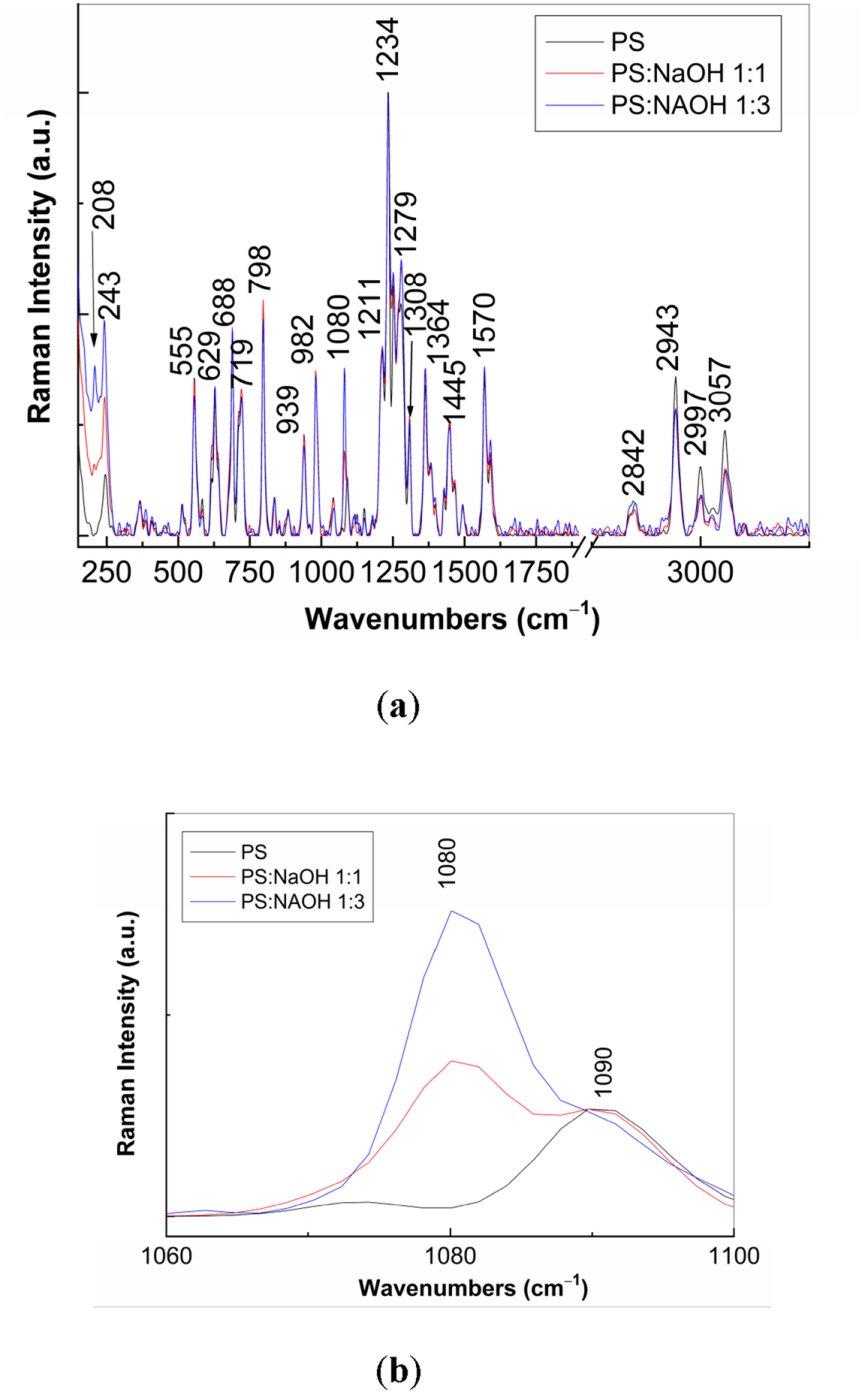
Raman spectra, in the 100–3500 cm^−1^ (**a**) and 1060–1100 cm^−1^ (**b**) spectral ranges, of PS (**1**) (curve black) and NaOH-reacted PS (**1**), when the weight ratio between PS (**1**) and NaOH was of 1:1 (red curve) and 1:3 (blue curve).

**Figure 13:**
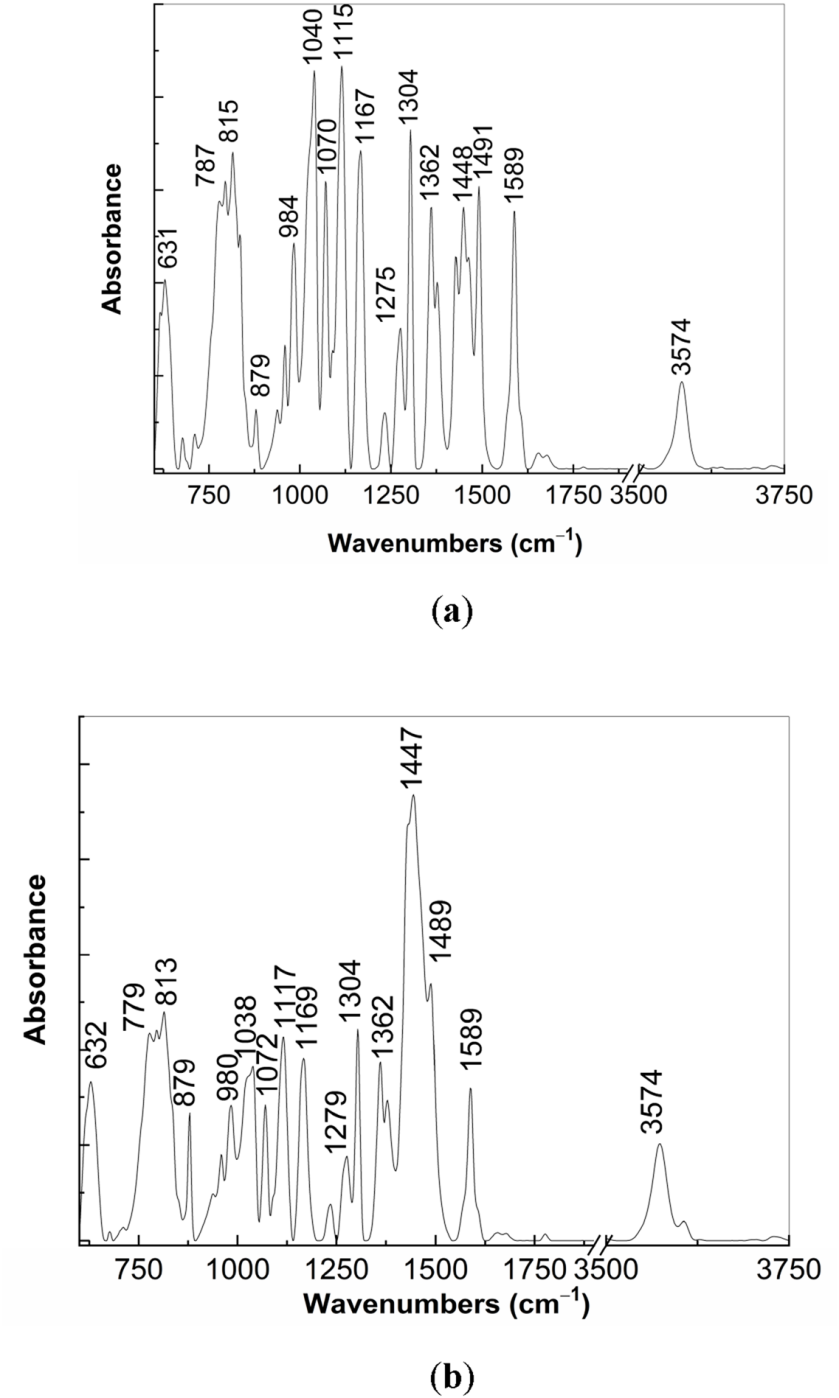
FTIR spectra of NaOH-reacted PS (**1**), when the weight ratio between PS (**1**) and NaOH is of 1:1 (**a**) and 1:3 (**b**).

**Figure 14:**
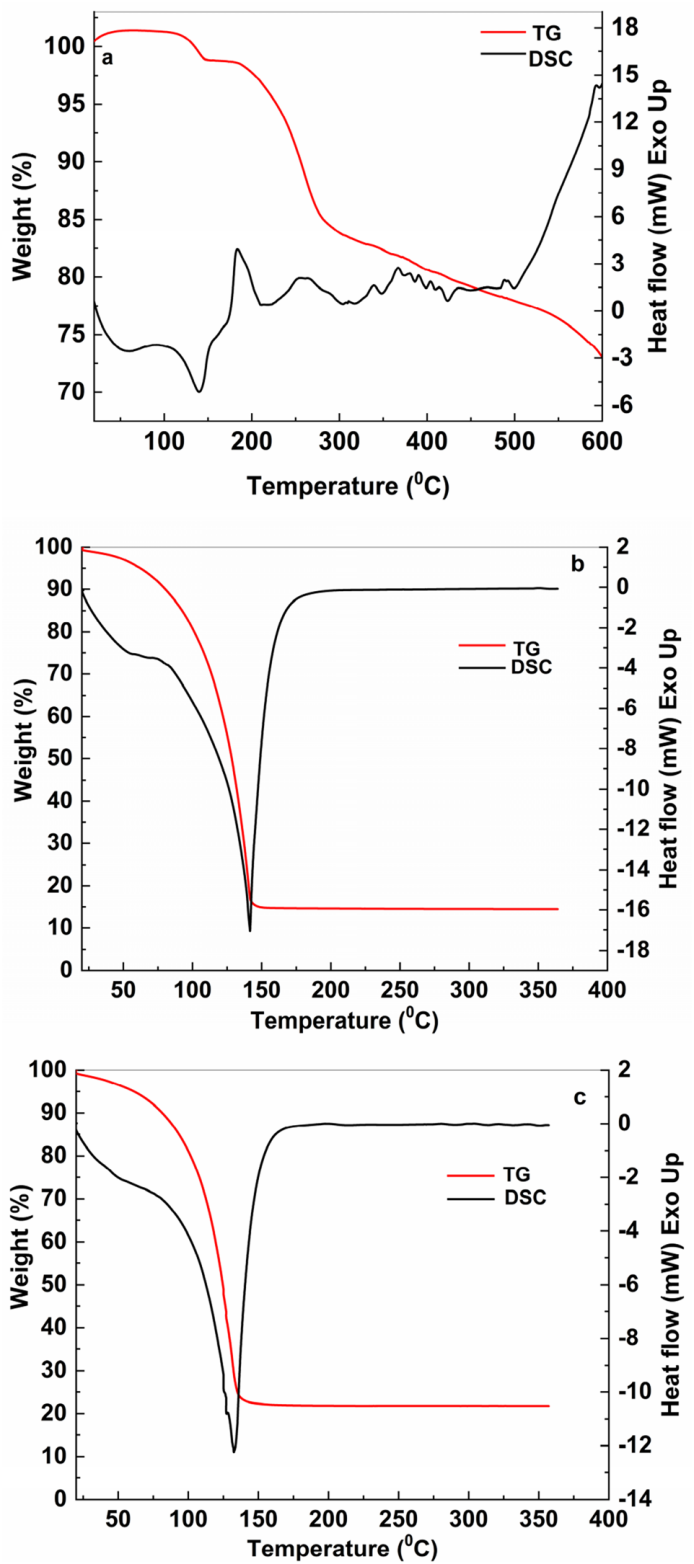
TG-DSC curves of PS in dark conditions (**a**) and the samples of PS (**b**) and NaOH-reacted PS (**1**) (**c**) after exposure to UV light.

**Figure 15:**
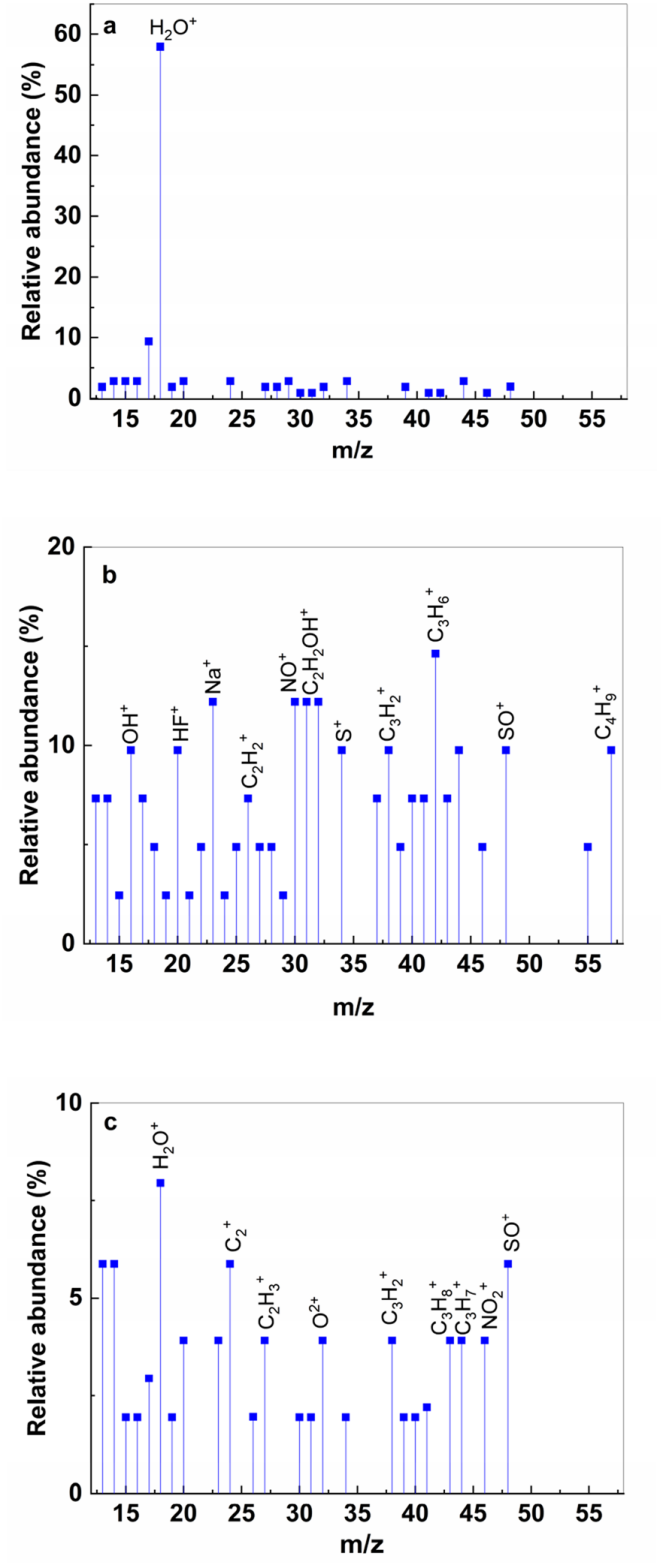
MS curves of PS (**1**) in dark conditions (**a**) and after exposure to UV light of PS (**1**) (**b**) and NaOH-reacted PS (**1**) (**c**).

In Fig. 9 is highlighted the behavior the PL spectra of the samples of PS (**1**) and Controloc (**4**) interacting with NaOH. Compared to Fig. 3a, the analysis of the Figs. 9a–c allows us to observe that, as increasing NaOH weight, an intensity growth of the PL spectrum of PS (**1**) from 2.5 × 10^5^ counts/sec (Fig. 3a) to 2.8 × 10^5^ counts/sec (Fig. 9a), 4.32 × 10^5^ counts/sec (Fig. 9b) and 4.17 × 10^5^ counts/sec (Fig. 9c), simultaneously with a shift of the PL band from 460 nm (Figs. 3a and 9a) to 456 nm (Fig. 9b) and 453 nm (Fig. 9c) take place. An analogue behavior is remarked for the PL spectra of Controloc (**4**) drug interacted with NaOH as follows: (i) the intensity growth of the PL spectra from 1.09 × 10^6^ counts/sec (Fig. 3b) to 2.1 × 10^6^ counts/sec (Fig. 9d); and (ii) a PL band shift from 446 nm (Fig. 3b) to 464 nm (Fig. 9d). Considering these variations, a difference between the PL band maximum of the Controloc (**4**) prior and after interaction, in dark condition, with the NaOH solution (1 ml, 0.3 M), is of 18 nm, while after the exposure to UV light this difference is of only of 2 nm. This result indicates that the products of photodegradation reaction of PS (**1**) show the common luminescent centers. The exposure of the samples to UV light has as effect a diminution of the PL band intensity of: (i) the samples of NaOH-reacted PS (**1**), when the volumetric ratio of the solutions of PS (**1**) 2 mg/ml and NaOH 0.3 M is of 2:1, 1.5:1.5 and 1:2, to ~ 2.19 × 10^5^ counts/sec (Fig. 9a), 2.68 × 10^5^ counts/sec (Fig. 9b) and 2.72 × 10^5^ counts/sec (Fig. 9c), respectively; and (ii) the Controloc (**4**) drug having as active compound PS (**1**; 2 mg/ml) that has interacted with the NaOH 0.3 M aqueous solution up to 7.5 × 10^5^ counts/sec (Fig. 9d). These variations indicate clearly that a chemical interaction under the assistance of UV light occurs both with H_2_O and NaOH. Above modifications reported in Fig. 9 are also accompanied of a modification in the profile of PL spectra. Figure 10 shows deconvolution of PL spectra of the sample resulted by the mixing of 1 ml PS (**1**) 2 mg/ml with 2 ml NaOH 0.3 M prior and after the 300 min UV light exposure. The PL spectrum shows three emission bands peaked at 2.41, 2.71 and 3.11 eV, whose intensities are equal to 1.41 × 10^5^, 4.14 × 10^5^ and 1.76 × 10^5^ counts/sec (Fig. 10a). After 300 min UV light exposure of the sample, the peaks of the three emission bands of Fig. 10b are situated to 2.35, 2.68 and 3.15 eV, their intensities being equal to 8.34 × 10^4^, 2.82 × 10^5^ and 1.37 × 10^5^ counts/sec, respectively. Summarizing these variations, the intensities decrease of the emission band at 2.41–2.35 and 2.75–2.68 eV (Fig. 10a,b) occurs simultaneously with intensity growth of the emission band peaked at 3.11–3.15 eV (Fig. 10a,b). To explain these facts, in the following additional information by UV–VIS spectroscopy, FTIR spectroscopy and Raman scattering are reported.

### The PS and Controloc photodegradation highlighted by the UV–VIS spectroscopy

**Scheme 2:**
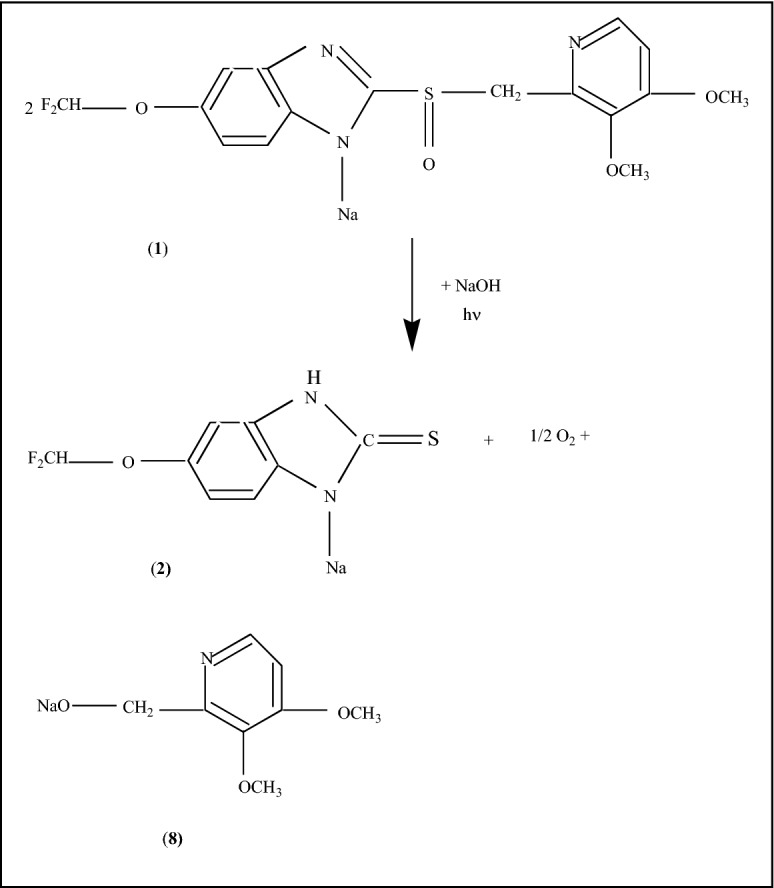
The photodegradation reactions of NaOH-reacted PS (**1**).

New compounds generate during the PS (**1**) exposure to UV light should induce the emergence of isosbestic points in the UV–VIS spectra. In support of this point of view, Fig. 11 shows the UV–VIS spectra of Controloc (**4**) drug, PS (**1**) alone and in the presence of NaOH. According to Figs. 11a,b, the UV–VIS spectra of PS (**1**) and the Controloc (**4**) drug show each a band with the maximum at 296 nm and 290 nm, respectively. These results are in good agreement with previous studies^2,4^. The exposure of the PS (**1**) solution to UV light, for 42 min., leads to: (i) a gradual absorbance diminution of the band at 296 nm, (ii) the emergence of another band at 246 nm and (iii) a slight widening of the band in the 325–450 nm spectral range, the variation that is accompanied of the presence of two isosbestic points at 262 nm and 306 nm (Fig. 11a). The UV light effect on the Controloc (**4**) solution is highlighted in Fig. 11b by: i) an important decrease in absorbance of ~ 61% of the band situated in the 250–325 nm spectral range, the change accompanied of a shift of the band from 290 to 276 nm and a broadening of the band in the 325–450 nm spectral range; and (ii) the emergence of two isosbestic points at 262 nm and 306 nm. These variations indicate clearly the new compounds formation during the exposure of the solutions of PS (**1**) and Controloc (**4**) to UV light, according to Scheme 1. According to Fig. 11c, NaOH-reacted PS (**1**) shows: (i) a band at 300 nm, prior to the UV light exposure; (ii) a band at 294 nm, whose absorbance gradually decreases as the exposure time to UV light growths up to 42 min.; and (iii) two isosbestic points at 270 nm and 320 nm. Scheme 2 shows reaction products of NaOH-reacted PS (**1**).

According to Scheme 2, the photodegradation products were 5-difluoromethoxy-3H-benzimidazole-2-thione sodium (**2**) and 2-oxymethyl- 3, 4-dimethoxypyridine sodium salt (**8**).

### New evidences for the reaction products of NaOH-reacted PS highlighted by Raman scattering and FTIR spectroscopy

To prove Scheme 2, vibrational properties of PS (1) and NaOH-reacted PS (1) prior and after the exposure to UV light are shown in Figs. 12 and 13. The reaction of PS (**1**) with NaOH, when the weight ratio of the two constituents is equal 1:1 and 1:3, leads to the appearance of a new line at 1080 cm^−1^, the intensity ratio of the Raman lines peaked at 1080 and 1090 cm^−1^ is equal to 1.45 and 2.85, respectively. The Raman line at 1080 cm^−1^ is assigned to the alkoxide group vibrational mode^22^. This Raman line has been also reported in the sodium salt of carboxylic compounds^23^. As increasing the NaOH weight used in the NaOH-reacted PS (**1**), in Fig. 12a new line at 208 cm^−1^ is observed. This line not belongs to NaOH. The early studies have reported that NaOH shows a Raman line at 215 cm^−1^^24^. In this stage, we are tempted to attribute the Raman line at 208 cm^−1^ to the ONa vibrational mode. The Raman line at 2842 cm^−1^ was reported to be attributed to the crystalline structure of PS dihydrate^25^. These variations confirm Scheme 2. Additional information concerning Scheme 2 are presented in Fig. 13. The reaction of PS (**1**) with NaOH involves changes in the IR spectra (Fig. 13) as follows: i) a down-shift of the IR bands from 797, 837 and 1450 cm^−1^ (Fig. 4b_1_) to 787, 815 and 1448 cm^-1^ (Fig. 13a) and 779, 813 and 1447 cm^−1^ (Fig. 13b); ii) an up-shift of the IR bands from 1112 and 1161 cm^−1^ (Fig. 4b_1_) to 1115 and 1167 cm^−1^ (Fig. 13a) and 1117 and 1169 cm^−1^ (Fig. 13b); iii) an absorbance decrease of the IR band at 1040 cm^−1^, indicating a lower weight of the SO and OSC bonds; iv) the absorbance increase of the IR bands localized in the 1300–1475 cm^−1^ spectral range so the A_1360_/A_1587_ and A_1491_/A_1587_ ratios are changed from 1.05 and 0.91 (Fig. 4b_1_) to 1.18 and 1.65, respectively (Fig. 13b); this result indicates a higher share of the OCCC and OCH bonds; and v) the presence of a new IR band at 3574 cm^−1^ (Figs. 13a,b) which often was assigned to the NH or OH stretching vibrational mode^21^. These results prove that the photodegradation products in Scheme 2 correspond to 5-difluoromethoxy-3H-benzimidazole-2-thione sodium (**2**) and 2-oxymethyl-3, 4-dimethoxy pyridine sodium salt (**8**).

### Correlated study of thermogravimetry and mass spectrometry

Figure 14 shows the TG-DSC curves of: (i) the PS prior the exposure to UV light and (ii) PS (**1**) and NaOH-reacted PS (**1**) after the exposure to UV light. According to Figs. 14b,c, the DSC curves of the PS and NaOH-reacted PS (**1**) after the exposure to UV light show pronounced endothermal peaks with maxima at ~ 140 ºC and ~ 132 ºC, respectively, associated with thermal decomposition processes of the two samples. These are accompanied by substantial mass losses of ~ 80% for both samples (TG curves). For the PS, a slow process in three stages of thermal decomposition is observed (TG curve) accompanied by a succession of endothermic and exothermic peaks with relatively low intensities visible on DSC curve (Fig. 14a). These can be associated with a complex process of decomposition of cycles corresponding to the structure of materials with the continuous formation of metastable phases that are decomposed as the temperature rises. Thus, it can be admitted that the presence of water and NaOH leads to a considerable decrease in the thermal stability of PS^26,27^. For a qualitative analysis of the gases resulting from the thermal decomposition processes of those three samples, a combination between thermal analysis and mass spectrometry (MS) was made^28^. Mass spectrometry (MS) has begun to be used more and more often over the past decade as an analytical method of analyzing drug development^29–31^. MS plays an essential role both in refining drug synthesis methods and in determining their purity and/or the presence of structural defects. This method is used in particular to highlight the mechanism of degradation by detecting and identifying the resulted compounds, or to follow the profile of the degradation product and to study the release of the drug^32,33^. In MS technique, the gases resulting from the thermal decomposition are ionized in a vacuum chamber and the charges and masses of the ions that break from samples are detected. The intensities of mass fragments as function of temperature resulted from sample decomposition are presented in Fig. 15. The probable molecules resulting from thermal decomposition are shown in Suppl. Table S3. For the assignment of fragments was used the NIST database library^33^. A good correlation of TG-DSC data with mass spectra is observed. The PS **(1)** in dark conditions sample is the most thermally stable and this is reflected in the mass spectrum by a low relative abundance of the registered molecular fragments, except for the water that evaporates in a larger quantity. The lower thermal stability of the other two samples analyzed is confirmed in the mass spectra by recording a large and varied number of molecular fragments corresponding to the thermal degradation mechanism.

## Conclusions

We have reported new results concerning the PS (**1**) photodegradation reaction. Using UV–VIS spectroscopy, photoluminescence, mass spectrometry, Raman scattering, thermogravimetry and FTIR spectroscopy the following conclusions are highlighted: (i) the emergence of the isosbestic points at 262–270 nm and 306–320 nm in the UV–VIS spectra of PS (**1**) which were correlated with the formation of new compounds of PS photodegradation in the presence of H_2_O and NaOH under UV light; (ii) the photodegradation process of PS (**1**) in the presence of H_2_O and O_2_ takes place in two stages, when a diminution of the weight of the CSCH and OSC bonds takes place, according to the studies of Raman scattering and FTIR spectroscopy; (iii) the reaction of PS (**1**) with NaOH results in 5-difluoromethoxy-3H-benzimidazole-2-thione sodium (**2**) and 2-oxymethyl- 3, 4-dimethoxypyridine sodium salt (**8**); (iv) the NH- bond in 5-difluoromethoxy-3H-benzimidazole-2-thione sodium (**2**) is highlighted by the presence of the IR band at 3547 cm^−1^; (v) the higher weight of the OCCC and OCH bonds in the NaOH-reacted PS proves the formation of 2-oxymethyl-3, 4-dimethoxypyridine sodium salt (**8**); vi) the presence of H_2_O and NaOH causes a considerable decrease in the thermal stability of PS; and vii) this work is the first which reports the key fragments resulted from thermal degradation of PS (**1**) as well as on PS in H_2_O and NaOH, respectively, after exposure to UV light.

## Methods

Pantoprazole sodium (PS (**1**), certified reference material, pharmaceutical secondary standard, purity 90.4%), NaH_2_PO_4_ (≥ 99.0%), Na_2_HPO_4_ × 12H_2_O (≥ 99.0%), NaOH (≥ 98%), Na_2_CO_3_ anhydrous (pharmaceutical primary standard grade), TiO_2_ (mixture of rutile and anatase, nanopowder < 100 nm particle size, 99.5% trace metals basis), sodium lauryl sulfate (SLS, certified reference material), polysorbate 80 and D-mannitol ((≥ 98.0%) were purchased from Sigma Aldrich, without other purifications. PB solutions with pH 8 and 6 were prepared in laboratory by mixing standard solutions of NaH_2_PO_4_ and Na_2_HPO_4_ × 12H_2_O.

The drug marketed under the name of Controloc (**4**) was bought from a local pharmacy. The composition of a tablet of Controloc was 20 mg PS, Na_2_CO_3_, mannitol, crospovidone, povidone K 90, calcium stearate, hypromellose, povidone K 25, titanium dioxide (E 171), yellow iron oxide (E172), propylene glycol, copolymer of ethyl acrylate and methacrylic acid (1: 1), polysorbate 80, sodium lauryl sulfate, triethyl citrate, shellac, iron oxide (E172), and concentrated ammonia.

The preparation of: (i) the solutions of PS in water or PB with pH = 6 and 8, having the concentration of 2 mg/ml, was carried by dissolving the PS powder by ultrasonication for 10 min.; and (ii) the Controloc (**4**) solutions was carried out by grounding a Controloc tablets in order to obtain a powder, which was dispersed in distilled water or in PB solutions by sonicated, time of 30 min., and then filtered. All solutions used in this work were prepared and stored in dark. In order to highlight the interaction of PS with alkaline medium, a stock solution of PS in water having the concentration of 2 mg/ml was mixed with a solution of NaOH 0.3 M and then ultrasonicated for 5 min. Using this protocol, three mixtures were prepared, the volumetric ratio of the PS and NaOH solutions being equal to: (i) 2:1; (ii) 1.5:1.5 and (iii) 1:2.

The influence of the excipients on the optical properties of PS was evaluated by preparing mixtures of PS (20 mg) in each of the excipients mentioned above (50 mg), the homogenization being performed by grinding for 5 min in dark conditions.

In order to evaluate the influence of water vapors and oxygen on the PS photodegradation process, the fluorescence cuvette containing 20 mg of PS (1) was purged with synthetic air and Ar 6.0, respectively, for 2 min, the photoluminescence spectra being successively recorded.

In all experiments, the samples were freshly prepared.

The photoluminescence (PL) and photoluminescence excitation (PLE) spectra of PS (**1**) and the Controloc (**4**) drug were recorded with a Fluorolog-3 spectrophotometer, FL-3.2.1.1 model, from Horiba Jobin Yvon, endowed with a Xe lamp having the power of 450 W. The studies of PL and PLE were carried out in the front face geometry, the excitation and the emission wavelengths were equal to 335 nm and 425 nm, respectively. In these studies, the excitation and emission slits were equal to 3 and the integrate time was of 0.5 s. The irradiation power was equal to 4.42 mW/cm^2^. The data collection was performed with the FluorEssence TM software. A fluorescence cuvette from Suprasil quartz, for the spectral range 200–2500 nm, with the chamber volume of 3500 μl, cell provided with gas purge system and closes the hermetic was used for this analysis.

UV–VIS spectra of PS (**1**) and the Controloc (**4**) drug were recorded with a UV–VIS-NIR spectrophotometer, Lambda 950 model, from Perkin Elmer. Hellma absorption cuvettes, from Suprasil quartz, for spectral range 200–2500 nm, having pathlength of 5 mm and chamber volume of 1750 μl was used in these experiments. The data collection was performed using the Perkin Elmer UV Win Lab software and a scan speed of 266.75 nm/min.

The vibrational properties of PS (**1**) and the Controloc (**4**) drug were recorded with: (i) a FT Raman spectrophotometer, Multiram model, in backscattering geometry, endowed with a YAG:Nd laser; the Raman spectra were recorded at the excitation wavelength equal to 1064 nm with a resolution of 1 cm^−1^, using the Opus 8.5 software; and (ii) a FTIR spectrophotometer, Vertex 80 model, in the geometry of attenuated total reflection, with a resolution of 2 cm^−1^, using the Opus 6.0 software. Both equipments were bought from Bruker. PS (**1**) in powder state was used as received for the Raman and FTIR spectroscopy studies. Vibrational properties of water-soluble PS photodegradation compounds were deposited on a Si plate and after evaporation of water, the film was used to record IR spectra in the 600–3500 cm^−1^ range.

The photodegradation study of PS interacting with alkaline medium were studied using the mixtures of PS and NaOH having the volumetric ratio equal to 1.5:1.5 and 1:2 from which 1 ml was taken and deposited onto a Si plate and dried at 100 °C under vacuum for 1 h. Crystallized powder resulted after drying was collected and used in the Raman and FTIR studies.

All samples containing PS were directly exposed to the UV light, using a mercury lamp emitting at 257 nm having the power of 350 W, which was purchased from Newport company. The irradiation power was equal to 4.46 mW/cm^2^.

Complex thermal investigations were performed on a SETARAM SETSYS Evolution 18 device in a thermogravimetry—differential scanning calorimetry (TG-DSC) thermal analyzer mode. Samples were measured in a flow (16 ml/min) of synthetic air (20% O_2 :_ 80% N_2_), with a heating rate of 5 ºC/min., from room temperature up to: (i) 380 ºC in the case of the samples of PS aqueous solution and the PS interacted with NaOH and (ii) 600 ºC in the case of the PS in powder state. The accuracy of heat flow measurements was ± 0.001 mW, and the temperature precision of ± 0.1 K. The gases resulted during the thermal measurements have been monitored with a QMS 301 OMNISTAR PFEIFFER mass spectrometer coupled at SETARAM device. The data were acquired in MID (Multiple Ion Detection) mode. In this configuration, gases were calculated by multiplying the intensities by previously determined calibration factors^34^. In this work, the MS data has been recorded through calibration of the ambient air species (argon, nitrogen and oxygen).

## Supplementary Information

Supplementary Information.
