# Supplementary material for: Correlated studies of photoluminescence, vibrational spectroscopy and mass spectrometry concerning the pantoprazole sodium photodegradation
Source: Sci Rep. 2022 Jun 9;12:9515. doi: 10.1038/s41598-022-13648-6 (PMC9184494; doi:10.1038/s41598-022-13648-6)
Supplement: Supplementary file 1 — Supplementary Information. [file 41598_2022_13648_MOESM1_ESM.docx]

**Supplementary Information**

**Correlated studies of photoluminescence, vibrational spectroscopy and mass spectrometry concerning the pantoprazole sodium photodegradation**

Mihaela Baibarac^1,*^, Mirela Paraschiv^1^, Radu Cercel^1^, Ion Smaranda^1^, Cristina Bartha^2^, Alexandru Trandabat^3^

^1^National Institute of Materials Physics, Laboratory of Optical Processes in Nanostructured Materials, Atomistilor Street 405A, POB MG 7, Bucharest 077125, Romania

^2^National Institute of Materials Physics, Laboratory of Magnetism & Superconductivity, Atomistilor Street 405A, POB MG-7, R077125, Bucharest, Romania

^3^SC Intelectro Iasi SRL, Iasi 700470, Romania

**Corresponding author**: Dr. M. Baibarac

E-mail: [barac@infim.ro](mailto:barac@infim.ro);

| ****  **a_1_** | ****  **a_2_** |
| --- | --- |
| ****  **b_1_** | ****  **b_2_** |
| ****  **c_1_** | ****  **c_2_** |
| ****  **d_1_** | ****  **d_2_** |
| ****  **e_1_** | ****  **e_2_** |
|  | ****  **f** |

**Fig. S1**. PL spectra of: PVP (a_1_), PS - PVP (a_2_), TiO_2_ (b_1_), PS - TiO_2_ (b_2_), SLS (c_1_), PS - SLS (c_2_), P80 (d_1_), PS - P80 (d_2_), D-mannitol (e_1_), PS - D-mannitol (e_2_), PS - Na_2_CO_3_ (f)

**a**

**b**

**Fig. S2**. Deconvolution of PL spectrum of PS in powder state, prior (**a**) and after 300 min of UV light exposure (**b**)

a

b

**Fig. S3**. The IR spectra of spectra of the mixture consisting of: (**a**) 2-thiomethyl-3, 4-dimethoxypyridine and 2-hydroxymethyl-3, 4-dimethoxypyridine and (**b**) 5-difluoromethoxy-3H-benzimidazole-2-thione sodium and 5-difluoromethoxy-3H-benzimidazole sodium.

| ****  **a_1_** | ****  **a_2_** |
| --- | --- |
| ****  **b_1_** | ****  **b_2_** |

**Fig. S4**. The PLE and PL spectra of the mixture consisting of: (**a_1_ and a_2_**)2-thiomethyl-3, 4-dimethoxypyridine and 2-hydroxymethyl-3, 4-dimethoxypyridine and (**b_1_ and b_2_**) 5-difluoromethoxy-3H-benzimidazole-2-thione sodium and 5-difluoromethoxy-3H-benzimidazole sodium.

| ****  **a_1_** | ****  **b_1_** |
| --- | --- |
| ****  **a_2_** | ****  **b_2_** |
| ****  **a_3_** | ****  **b_3_** |
| ****  **a_4_** | ****  **b_4_** |
| ****  **a_5_** | ****  **b_5_** |

**Fig. S5**. PL spectra of PS in the presence of air synthetic (**a_1_**) and Ar (**b**_1_); prior to exposure to UV light (red curve) and after 300 min UV light exposure (magenta curve). Black curves in Figures (**a_1_**) and (**b_1_**) correspond to intermediate PL spectra. Red and black curves in Figures (**a_2_**) and (**b_2_**) show PL spectra of PS in the presence of air synthetic and Ar, prior and after to UV light exposure for 48 min or 23 min, respectively. Deconvolution of PL spectra of PS in the presence of air synthetic and Ar: prior (**a_3_** and **b**_3_) and after UV light exposure for 48 min and 23 min, respectively (**a_4_** and **b_4_**). Figures **a**_5_ and **b_5_** show deconvolution of PL spectra of PS in the presence of air synthetic and Ar, respectively, after 300 min of the UV light exposure.

**Table S1**. Experimental vibration wavenumbers (ν, cm^-1^) in the Raman spectra of PS (**1**)

| ν, cm^-1^ | **Assignment of vibrational mode [12, 13]** |
| --- | --- |
| 243-555 | out-of-plane bending OSCC |
| 629 | in plane bending CNC |
| 688 | stretching NC |
| 719 | torsion NCSC |
| 798 | stretching CC + in plane bending COC |
| 939 | stretching CH |
| 982 | stretching CH + torsion CSCH |
| 1090 | stretching CF |
| 1211 | in plane bending HCH |
| 1234 | stretching OC + in plane bending HCC + torsion COCC |
| 1271 | out-of-plane bending CNC |
| 1308 | stretching CC |
| 1364 | out-of-plane OCCC |
| 1445 | stretching CH + torsion HCCC |
| 1570 | out-of-plane bending SCNN |
| 2943 | out-of-plane of the C-H bonds in CH CH_3_ groups |
| 3000 | out-of-plane of the C-H bonds in CH_2_ CH_3_ groups |
| 3057 | out-of-plane of the C-H bonds in CH_3_ groups |

**Table S2**. Experimental vibration wavenumbers (ν, cm^-1^) in the IR spectra of PS (**1**)

| ν, cm^-1^ | **Assignment of vibrational mode [12,13]** |
| --- | --- |
| 629 | in plane bending CNC |
| 677 | stretching NC |
| 797 | stretching CC + in plane bending COC |
| 837 | in plane bending HCC |
| 883 | stretching CH |
| 984 | stretching CH + torsion CSCN |
| 1040 | stretching OC + stretching SO + in plane bending OSC |
| 1070 | stretching CF |
| 1112 | out-of-plane OCFF |
| 1161 | in plane bending NCH + HCC |
| 1275 | out-of-plane bending NCC |
| 1304 | stretching CC |
| 1360 | out-of-plane bending OCCC |
| 1450 | stretching CH+ torsion HCCC |
| 1491 | torsion COCH |
| 1587 | in plane bending CCN |

**Table S3**. Spectral peaks of the pyrolysis of the three samples and their key fragments

| **Samples** | **Mass number (m/z)** | **Relative abundance** | **Key fragments** | **Probable** |
| --- | --- | --- | --- | --- |
| **PS** | 13 | 1.87 % | CH+ | CH |
|  | 14 | 2.80 % | N^+^ | N_2_ |
|  | 15 | 2.80 % | CH_3_^+^  NH^+^ | CH_3_  NH |
|  | 16 | 2.80 % | O^+^ | O_2_ |
|  | 17 | 9.35 % | OH^+^ | H_2_O |
|  | 18 | 57.94 % | H_2_O^+^ | H_2_O |
|  | 19 | 1.87 % | F^+^ | F_2_ |
|  | 20 | 2.80 % | HF^+^ | HF |
|  | 23 | 1.87 % | Na^+^ | Na |
|  | 24 | 2.80 % | C^2+^ | C_x_H_y_ |
|  | 27 | 1.87 % | C_2_H_3_^+^ | C_2_H_3_ |
|  | 28 | 2.57 % | N^2+^ | N_2_ |
|  | 29 | 1.87 % | C_2_H_5_^+^ | C_2_H_5_ |
|  | 30 | 0.93% | NO^+^ | NO_2_ |
|  | 31 | 1.91 % | C_2_H_2_OH^+^ | C_2_H_2_OH |
|  | 32 | 1.87 % | O^2+^ | O_2_ |
|  | 34 | 2.80 % | S^+^ | H_2_S |
|  | 39 | 1.87 % | C_3_H_3_^+^ | C_3_H_2_ |
|  | 41 | 0.93 % | C_3_H_5_^+^ | C_3_H_5_ |
|  | 42 | 0.93 % | C_3_H_6_^+^ | C_3_H_6_ |
|  | 44 | 2.80 % | CO^2+^  N_2_O^+^  C_3_H_8_^+^ | CO_2_  N_2_O  C_3_H_8_ |
|  | 46 | 0.93 % | NO_2_^+^ | NO_2_ |
|  | 48 | 1.93 % | SO^+^ | SO_2_ |
| **PS reacted with NaOH** | 13 | 5.88 % | CH^+^ | CH |
|  | 14 | 5.88% | CH_2_^+^  N^+^ | CH_2_  N_2_ |
|  | 15 | 1.96 % | CH_3_^+^  NH^+^ | CH_3_  NH |
|  | 16 | 1.96% | O^+^  CH_4_^+^  NH_2_^+^ | O_2_  CH_4_  NH_3_ |
|  | 17 | 2.94 % | OH^+^  NH^3+^ | H_2_O  NH_3_ |
|  | 18 | 7.95 % | H_2_O^+^ | H_2_O |
|  | 19 | 1.96% | F^+^ | F_2_ |
|  | 20 | 3.92% | HF^+^ | HF |
|  | 23 | 3.92% | Na^+^ | Na |
|  | 24 | 5.88% | C^2+^ | C_x_H_y_ |
|  | 26 | 1.97% | C_2_H_2_^+^ | C_2_H_2_ |
|  | 27 | 3.92% | C_2_H_3_^+^ | C_2_H_3_ |
|  | 30 | 1.96% | C_2_H_6_^+^  NO^+^ | C_2_H_6_  NO |
|  | 31 | 1.96% | C_2_H_2_OH^+^ | C_2_H_2_OH |
|  | 32 | 3.92% | O^2+^ | O_2_ |
|  | 34 | 1.96% | S^+^ | H_2_S |
|  | 38 | 3.92% | C_3_H_2_^+^ | C_3_H_2_ |
|  | 39 | 1.96% | C_3_H_3_^+^ | C_3_H_2_ |
|  | 40 | 1.96% | C_3_H_4_^+^ | C_3_H_4_ |
|  | 41 | 2.21% | C_3_H_5_^+^ | C_3_H_5_ |
|  | 43 | 3.92% | C_3_H_7_^+^ | C_3_H_6_ |
|  | 44 | 3.92% | CO^2+^  N_2_O^+^  C_3_H_8_^+^ | CO_2_  N_2_O  C_3_H_8_ |
|  | 46 | 3.92% | NO_2_^+^ | NO_2_ |
|  | 48 | 5.88% | SO^+^ | SO_2_ |
| **PS in H_2_O** | 13 | 7.32% | CH^+^ | CH |
|  | 14 | 7.32% | CH^2+^ | CH_2_ |
|  | 15 | 2.44% | CH^3+^  NH^+^ | CH_3_  NH |
|  | 16 | 2.44% |  |  |
|  | 17 | 7.32% | OH^+^  NH_3_^+^ | H_2_O  NH_3_ |
|  | 18 | 4.88% | H_2_O^+^ | H_2_O |
|  | 19 | 2.44% | F^+^ | F_2_ |
|  | 20 | 9.76% | HF^+^ | HF |
|  | 23 | 12.20% | Na^+^ | Na |
|  | 24 | 2.44% | C_2_^+^ | C_x_H_y_ |
|  | 25 | 4.88% | C_2_H^+^ | C_2_H |
|  | 26 | 7.32% | C_2_H_2_^+^ | C_2_H_2_ |
|  | 27 | 4.88% | C_2_H_3_^+^ | C_2_H_3_ |
|  | 28 | 4.88% | N_2_^+^  CO^++^ | N_2_  CO |
|  | 29 | 2.44% | C_2_H_5_^+^ | C_2_H_5_ |
|  | 30 | 12.20% | C_2_H_6_^+^  NO^+^ | C_2_H_6_  NO |
|  | 31 | 12.20% | C_2_H_2_OH^+^ | C_2_H_2_OH |
|  | 32 | 12.20% | O^2+^ | O_2_ |
|  | 34 | 9.76% | S^+^ | H_2_S |
|  | 37 | 7.32% |  |  |
|  | 38 | 9.76% | C_3_H_2_^+^ | C_3_H_2_ |
|  | 39 | 4.88% | C_3_H_3_^+^ | C_3_H_2_ |
|  | 40 | 7.32% | C_3_H_4_^+^ | C_3_H_4_ |
|  | 41 | 7.32% | C_3_H_5_^+^ | C_3_H_5_ |
|  | 42 | 14.63% | C_3_H_6_^+^ | C_3_H_6_ |
|  | 43 | 7.32% | C_3_H_7_^+^ | C_3_H_7_ |
|  | 44 | 9.76% | CO^2+^  N_2_O^+^  C_3_H_8_^+^ | CO_2_  N_2_O  C_3_H_8_ |
|  | 46 | 4.88% | NO_2_^+^ | NO_2_ |
|  | 48 | 9.76% | SO^+^ | SO_2_ |
|  | 55 | 4.88% | C_4_H_7_^+^ | C_4_H_7_ |
|  | 57 | 9.76% | C_4_H_9_^+^ | C_4_H_9_ |
